# Mechanisms of action of ionic liquids on living cells: the state of the art

**DOI:** 10.1007/s12551-020-00754-w

**Published:** 2020-09-16

**Authors:** Pallavi Kumari, Visakh V.S. Pillai, Antonio Benedetto

**Affiliations:** 1 Department of Sciences, University of Roma Tre, 00146 Rome, Italy; 2 School of Physics, University College Dublin, Dublin 4, Ireland; 3 Conway Institute of Biomolecular and Biomedical Research, University College Dublin, Dublin 4, Ireland; 4 Laboratory for Neutron Scattering, Paul Scherrer Institute, 5232 Villigen, Switzerland

**Keywords:** Ionic liquids, Mechanism of action, Cell membrane viscoelasticity, Cell membrane disruption, Nuclear membrane disruption, Mitochondrial permeabilization, Mitochondrial dysfunction, Chloroplast damage, DNA damage, Transmembrane protein function, Reactive oxygen species, Signaling pathways, System biology

## Abstract

Ionic liquids (ILs) are a relatively new class of organic electrolytes composed of an organic cation and either an organic or inorganic anion, whose melting temperature falls around room-temperature. In the last 20 years, the toxicity of ILs towards cells and micro-organisms has been heavily investigated with the main aim to assess the risks associated with their potential use in (industrial) applications, and to develop strategies to design greener ILs. Toxicity, however, is synonym with affinity, and this has stimulated, in turn, a series of biophysical and chemical-physical investigations as well as few biochemical studies focused on the mechanisms of action (MoAs) of ILs, key step in the development of applications in bio-nanomedicine and bio-nanotechnology. This review has the intent to present an overview of the state of the art of the MoAs of ILs, which have been the focus of a limited number of studies but still sufficient enough to provide a first glimpse on the subject. The overall picture that emerges is quite intriguing and shows that ILs interact with cells in a variety of different mechanisms, including alteration of lipid distribution and cell membrane viscoelasticity, disruption of cell and nuclear membranes, mitochondrial permeabilization and dysfunction, generation of reactive oxygen species, chloroplast damage (in plants), alteration of transmembrane and cytoplasmatic proteins/enzyme functions, alteration of signaling pathways, and DNA fragmentation. Together with our earlier review work on the biophysics and chemical-physics of IL-cell membrane interactions (Biophys. Rev. 9:309, 2017), we hope that the present review, focused instead on the biochemical aspects, will stimulate a series of new investigations and discoveries in the still new and interdisciplinary field of “ILs, biomolecules, and cells.”

## Introduction

### Ionic liquids

**Fig. 1:**
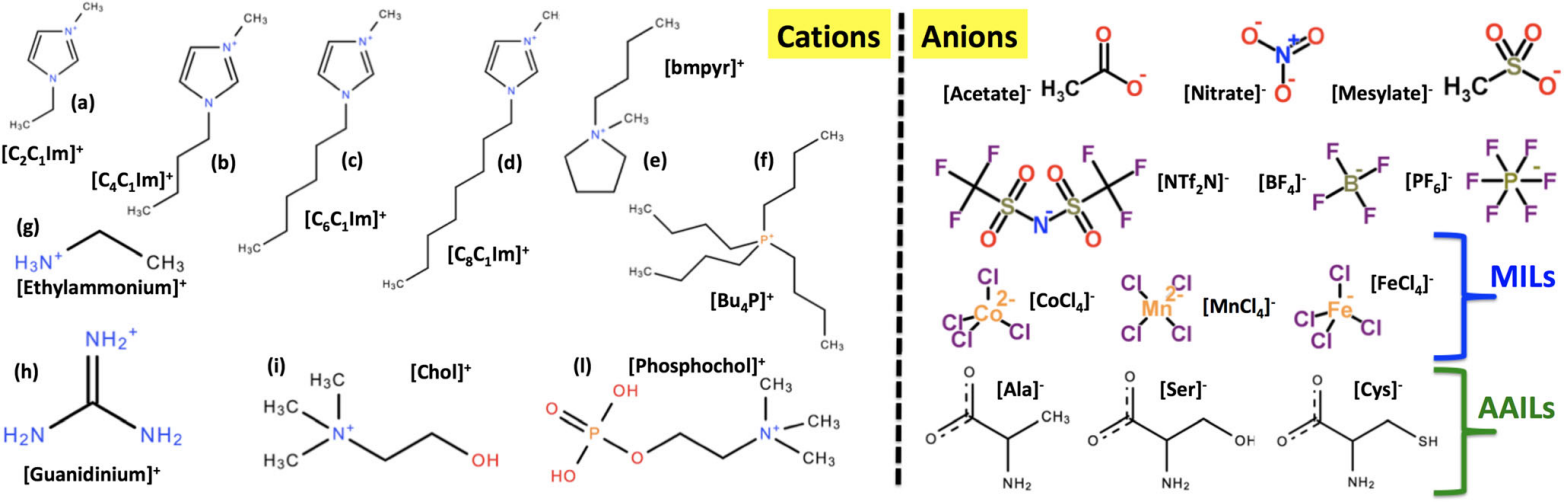
Cations and anions from selected and common ILs. The MIL (magnetic IL) subcategory consists of magnetic anions; the AAIL (amino acid IL) subcategory consists of anions made of deprotonated amino acids. Figure was taken from Benedetto and Ballone (2018a) and reproduced with permission from the publisher

Ionic liquids (ILs) are ionic compounds composed by an organic cation and either an organic or inorganic anion (Fig. 1), which possess several interesting properties such as being liquid around room temperature and having low vapor pressure (Welton 1999; Hallett and Welton 2011). Historically, the first IL has been probably discovered in 1914 by Paul Walden, followed then in 1951 by Hurley and Weir. However, the research field of ILs came to existence as a such only in the 1980s and consolidate later in the 1990s: one of the most common families of ILs, for example, that is the imidazolium-based ILs family, has been discovered in the 1980s by John Wilkes, and only by the end of the twentieth century, ILs were coming to the attention of a wider audience (Welton 2018). At this stage, the extreme flexibility in designing ILs became clear and, in turn, the potentially huge variety of these organic ionic compounds, which started to be investigated beyond the historical chemical community who synthesized them (Bai et al. 2019). Because the first ILs showed a clear green character, there was the assumption, if not the hope, that this could be extended to all the ILs (Petkovic et al. 2011; Earle and Seddon 2000). However, in the 2000s, several biological studies highlighted a moderate-to-high toxicity of ILs towards several micro-organisms, plants, cell lines, and potentially humans (Bernot et al. 2005; Pretti et al. 2006; Pham et al. 2008; Studzińska and Buszewski 2009; Matzke et al. 2009; Li et al. 2009; Coleman and Gathergood 2010; Frade and Afonso 2010; Costa et al. 2017; Bubalo et al. 2017; Thamke et al. 2019; Pawłowska et al. 2019; Leitch et al. 2020a; Leitch et al. 2020b). For example, ILs based on amino acids (Benedetto et al. 2014a), which were supposed to be more bio-friendly, turned out to have higher toxicity level than their common counterparts (Egorova et al. 2015).

### Toxicity of ILs

Several works and few reviews have been published in the last 15 years on the toxicity of ILs. Overall, the main aim of these works was to highlight the moderate-to-high toxicity of ILs, pointing the potential danger in their use in industrial applications and suggesting how to tune their chemical structures to reduce toxicity. Typically, the EC50 value of ILs, which is the IL-concentration for which the cell viability reduces by half, is in the micromolar-to-millimolar range. For example, it has been reported that in the case of rat pheochromocytoma PC12 cells incubated in imidazolium-based ILs, the EC50 oscillates from > 10 mM to 24 μM after 24 h incubation, respectively, in [C_4_mim][Br] and [C_12_mim][Br], and that the cell viability decreases by IL-concentration and IL-chain length (Li et al. 2012a). However, it is important to note that EC50 values in the nanomolar range have also been observed (Malhotra et al. 2014). It has been shown that the toxicity depends mainly on IL-cations with, in general, a minor impact of IL-anions (Stolte et al. 2006). Usually, the effect of anions is moderate in the sense that it does not change the order of magnitude of the EC50; however, few exceptions have been observed. For example, it has been shown that aureate anions reduce the EC50 by three orders of magnitude (Sioriki et al. 2019). In the same study, it has been pointed out that unsaturated-ring imidazolium-based ILs have lower EC50 than saturated cases. It is generally accepted that IL toxicity increases with increasing IL-concentration and IL-chain length (Ranke et al. 2004). In particular, the correlation between IL toxicity and the degree of IL-cation lipophilicity has been highlighted in several studies (Ranke et al. 2006; Ranke et al. 2007; Vraneš et al. 2019), suggesting membrane damage as a potential mechanism of toxicity. In this respect, it is important to mention that few exceptions exist (Wang et al. 2015; Drücker et al. 2017). It has also been shown that IL toxicity can be time-dependent (Li et al. 2015); this itself suggests already the existence of other biochemical mechanisms of toxicity in addition to the membrane damage mechanism. The effect of mixing two ILs has also been investigated. In this context, toxicity of mixtures of one IL with another IL or with other salts has been investigated for the human cervical carcinoma epithelial cell line HeLa (Wang et al. 2007). Very recently, Ananikov and co-workers studied cytotoxicity of aqueous solutions of binary mixtures of common ILs and showed that it mostly did not comply with the concentration addition model, suggesting the occurrence of toxicity-affecting interactions. They observed antagonistic effects in the studied systems, and the formation of micro-heterogeneous water structures in both single-component and two-component IL-solutions (Kashin et al. 2016; Egorova et al. 2020). To a more complete picture of the toxicity of ILs, please refer to one (or more) of the several reviews published in the last decade, which would represent a very useful starting point for researchers interested in gaining an overview of this field of study (Pham et al. 2010; Egorova and Ananikov 2014; Kudłak et al. 2015; Heckenbach et al. 2016; Sivapragasam et al. 2020).

### The “positive side” of IL toxicity

Whereas the discovery of the toxicity of ILs has broken the dreams of several researchers, it stimulated, on the other hand, a series of new studies interested in taking advantage of the affinity of ILs towards cells for applications in pharmacology, bio-medicine, and bio-nanotechnology (Hough et al. 2007; Carson et al. 2009; Stoimenovski et al. 2012; Marrucho et al. 2014; Williams et al. 2014; Egorova et al. 2017; Sahbaz et al. 2017; Agatemor et al. 2018; Benedetto and Ballone 2018a; Tanner et al. 2020; Shi et al. 2020). For example, it has been shown that the oral delivery of a choline-based IL prevents the absorption of fat molecules through intestinal tissue in rats (Nurunnabi et al. 2019). Apart from few very ad hoc “system biology” studies, as the one just mentioned, there are several studies highlighting the specificity of ILs towards cancer cells and their ability to kill those cells, leaving healthy cells almost unaffected (Kumar and Malhotra 2009; Malhotra and Kumar 2010; Wang et al. 2015; Stromyer et al. 2020). In this context, it has been shown, for example, that tri-*n*-butyl-*n*-hexadecyl-phosphonium bromide—a phosphonium-based IL with a halogen anion and balanced lengths of C_4_ and C_16_ alkyl chains—is approximately 100 times more toxic against HeLa cells than several ammonium-based ILs, but it remains inactive against human chronic myelogenous leukemia K562 cells; complementarily, triphenyl-alkyl-phosphonium iodides, with shorter C_1_ and C_5_ alkyl chains, resulted to be inactive against HeLa cells, but very active against K562 cells (Bachowska et al. 2012). More recently, (i) a novel betulinic acid–derived IL, i.e., [P_6,6,6,14_][BA], has shown a specific cytotoxicity against human hepatocellular carcinoma HepG2 and human lung carcinoma A459 tumor cell lines (Silva et al. 2019), and (ii) a new imidazolium IL with a triphenylphosphonium substituent, applied intravesically to mouse, has selectively killed bladder cancer cells, leaving adjacent healthy cells unaffected (Stromyer et al. 2020). This selective ability of ILs together with their extreme flexibility in design makes them potential candidates against cancers. Moreover, non-toxic ILs or sub-toxic IL-doses are also showing promising applications. For example, it has been shown that some ammonium-based ILs, which are not toxic against HeLa and K562 cells, can be then used as gene delivery vehicles since they could electrostatically interact with negatively charged DNA or RNA molecules (Bachowska et al. 2012). In the same context, more recently, the ability of a novel di-cationic imidazolium IL in gene transfer has been shown: cell culture experiments have revealed that mixed liposomes containing this novel double-tail lipid-mimic IL can serve as plasmid DNA delivery vehicles (Paulisch et al. 2020). Toxicity of ILs towards both Gram-positive and Gram-negative bacteria has also been observed, and associated to the ability of ILs to disrupt bacterial membranes, supported by the similarity in the structure of ILs with detergents, pesticides, and antibiotics; moreover, some ILs exhibit structural similarity with cationic surfactants which can disrupt membrane-bound proteins (Docherty and Kulpa Jr. 2005; Carson et al. 2009; O’Toole et al. 2012; Zakrewsky et al. 2014; Ganapathi and Ganesan 2017; Ibsen et al. 2018). Finally, it is also interesting to mention here that ILs possess antiviral activity. In this respect, it has been found that some ILs can stop the replication cycle of the human immunodeficiency virus (HIV) by inhibiting the function of HIV-integrase, which is an important enzyme in the virus replication cycle (Maddali et al. 2011). The viricidal activity of ILs has been proven in few other cases, but the associated mechanisms need further investigations (Kumar and Malhotra 2008; Sommer et al. 2018).

However, even though there are a relevant number of studies on the toxicity of ILs (as well as few nice and comprehensive reviews), only very few studies focus on the mechanisms/modes of action (MoAs) of these organic electrolytes. The knowledge of such mechanisms is very important not only to develop and design greener ILs, as the majority of the researchers in this field have been highlighting, but also to facilitate and tune the development of applications of ILs in bio-nanotechnology and bio-nanomedicine. This review has been written with the aim to gather together and facilitate the comparison of these MoAs of ILs; in this sense, it has the goal to present the state of the art of the MoAs of ILs.

### Biophysical and chemical-physical investigations of IL-biomembrane interactions

Based on our biophysical and chemical-physical backgrounds, we would like to mention that the toxicity of ILs has also stimulated several biophysical and chemical-physical investigations on the interaction between ILs and biomolecules. For example, it has been shown that ILs have the ability to interact with proteins and enzymes (Kumar and Venkatesu 2014; Kumar et al. 2017; Takekiyo and Yoshimura 2018; Pillai and Benedetto 2018; Bui-Le et al. 2020; Bharmoria et al. 2020) and with DNA and RNA (Tateishi-Karimata and Sugimoto 2018). Particular attention has been devoted to the investigation of the interaction between ILs and model cell membranes, with a special focus on phospholipid bilayers used as basic/first-order models of cell membranes (Benedetto 2017; Wang et al. 2018). This set of investigations, in particular, has been motivated by the observation/intuition that the interaction between ILs and cells has to be mediated/filtered by the cell membrane first, which represents the physical barrier that any foreign species has to pass/interact with before getting into the cell, and by the proven correlation between toxicity and lipophilicity of ILs (Ranke et al. 2006; Ranke et al. 2007; Vraneš et al. 2019). A palette of very diverse experimental techniques as well as computational methods have been employed, including neutron and X-ray scattering, atomic force microscopy (AFM), fluorescence techniques, and molecular dynamics simulations (Evans 2008; Benedetto et al. 2014a; Benedetto et al. 2014b; Yoo et al. 2014; Benedetto et al. 2015; Yoo et al. 2016a; Yoo et al. 2016b; Wang et al. 2016; Kontro et al. 2016; Drücker et al. 2017; Bhattacharya et al. 2017; Witos et al. 2017; Bornemann et al. 2020). In this respect, quasi-elastic neutron scattering has been employed to determine the effect on lipid diffusion due to the presence of IL-cations into the lipid region, showing, for example, a reduction of liver lipid lateral motion in the presence of [C_10_mim][BF_4_] (Sharma et al. 2017; Sharma and Mukhopadhyay 2018; Bakshi et al. 2020). In this context, neutron scattering, in tandem with computer simulations, can be a powerful and unique tool to investigate the chemical-physics of these systems (Magazù et al. 2008; Magazù et al. 2010; Magazù et al. 2012; Nandi et al. 2017; Benedetto and Ballone 2018b). Overall, the aim of these chemical-physics studies was (and still is) to understand the microscopic mechanisms of interaction between ILs and cell membranes, which represent a key step for the development of applications (Benedetto and Ballone 2018a; Kumari et al. 2020). It has emerged that the affinity of ILs towards phospholipid bilayers (i.e., lipophilicity) increases as a function of the hydrocarbon chain length of the cations as well as their toxicity. It has been shown that ILs can disrupt phospholipid bilayers as easier as longer is their cation hydrocarbon chain. This disruption occurs at IL-concentrations that are inversely proportional to the hydrocarbon chain length of the IL-cations, and usually in the range of the IL critical micellar concentration (CMC), which is the concentration of an IL in solution starting from which the IL is able to rearrange themselves and create super-molecular structures such as micelles and vesicles (Blesic et al. 2007; Yoo et al. 2016a; Yoo et al. 2016b). It has then been proposed that it is the presence of these super-molecular structures of ILs in solutions at the cell-water interface that drives the overall disruption of the cell membrane.

**Fig. 2:**
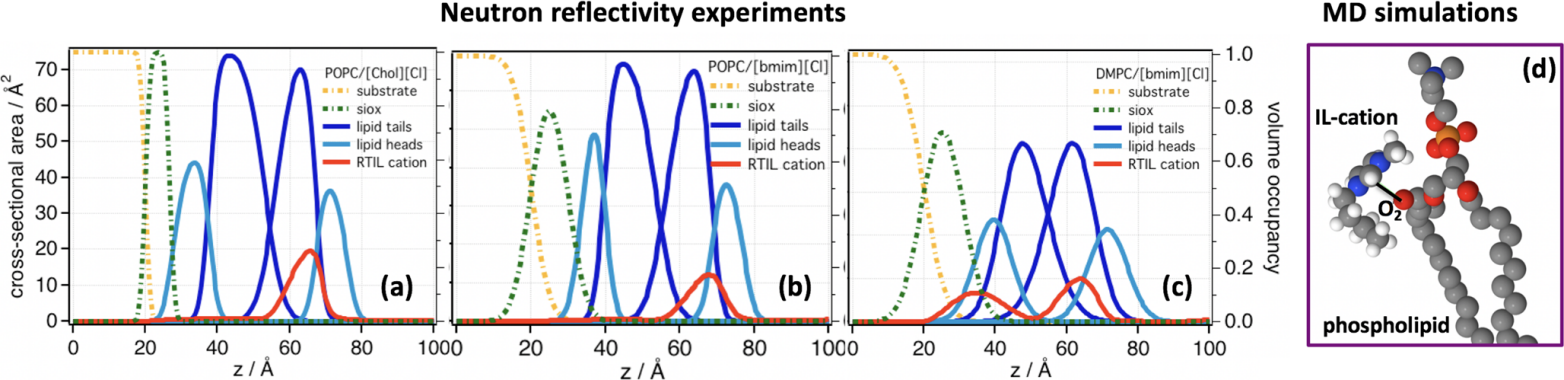
**a**–**c** Density distribution profiles as a function of height *z* from the surface of the substrate obtained by fitting the neutron reflectivity data taken from Benedetto et al. (2014b). Neutron reflectometry has allowed to model each single supported phospholipid bilayers with four different density distributions accounting for: (i) the inner lipid heads layer (cyan); (ii) the inner lipid tail layer (blue); (iii) the outer lipid tail layer (blue); (iv) the outer lipid heads layer (cyan); and also (v) the density distribution of the cations (red), whereas the anion (Cl^−^) is almost invisible to neutrons. Three cases are here reported where two different phospholipid bilayers interact with aqueous solutions of two different ILs at 0.5 M: **a** POPC and [Chol][Cl], **b** POPC and [C_4_mim][Cl], and **c** DMPC and [C_4_mim][Cl]. IL-cation absorption accounts for 8%, 6.5%, and 11% of the lipid bilayer volume, respectively. In **c**, the diffusion of the cations into the inner leaflet is apparent, and this can imply diffusion into the cytoplasm through the cellular lipid membrane. In **d**, a representative molecular dynamics simulations configuration of the [C_4_mim]^+^ IL-cation in close contact with a POPC molecule taken from Benedetto et al. (2015). Figures reproduced with permission from the publishers

**Fig. 3:**
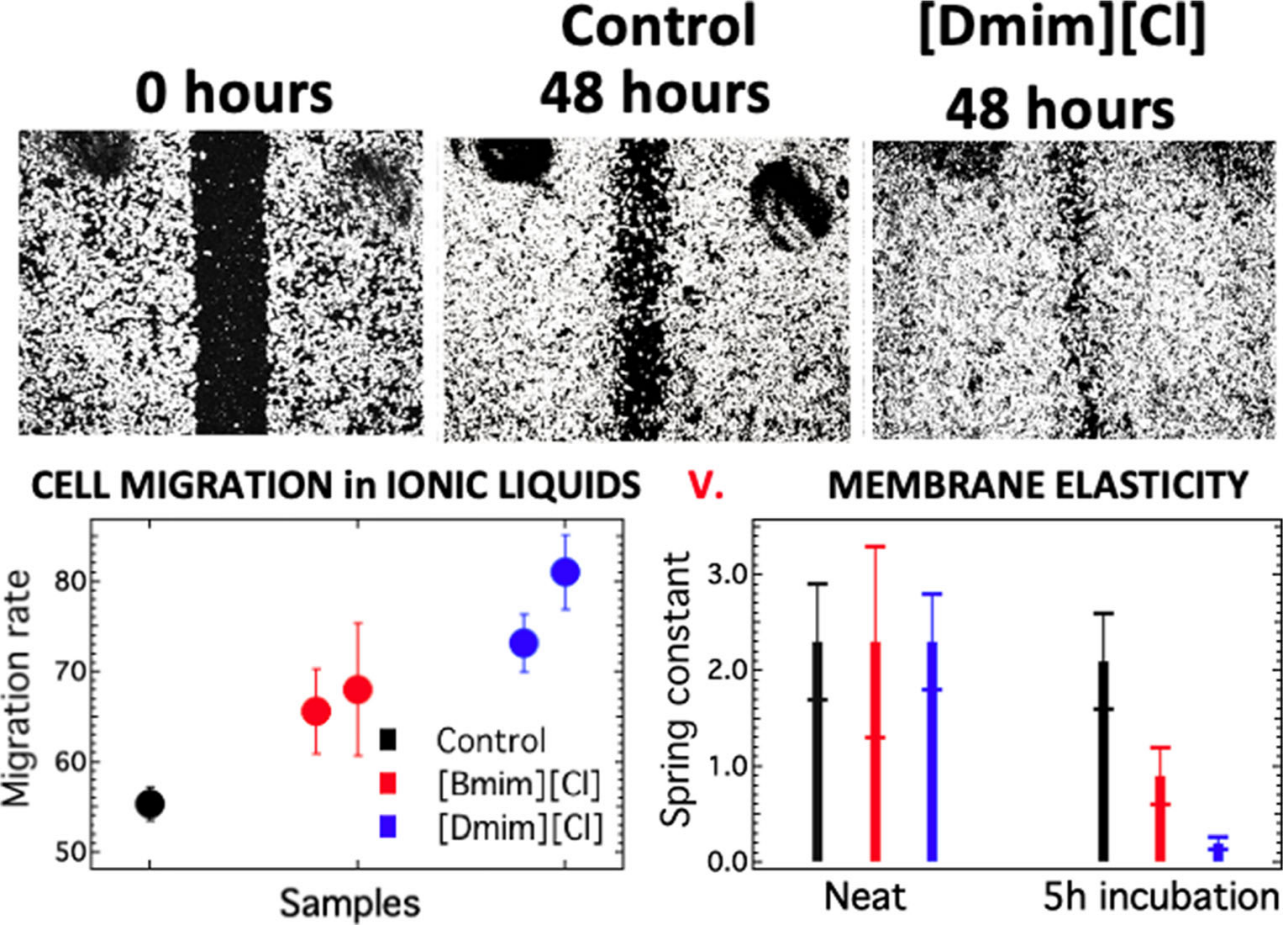
Cell migration and cellular lipid membrane elasticity for MDA-MB-231 cells incubated at sub-toxic concentrations of imidazolium ILs showing a correlation/relationship between the ability of ILs to reduce the cell membrane elasticity and their ability to enhance cell migration. Taken from Kumari et al. (2020) and reproduced with permission from the publisher

Generally speaking, the majority of the biochemical and chemical-physical studies on the interaction between ILs and model cell membranes have been designed with the aim to link cell death to membrane-IL interactions, actually and more precisely, to the membrane disruption by ILs. However, the prospective of a niche sub-class of these chemical-physical studies has been somehow different. Our focus, for example, has been on the effect of low doses of ILs on model biomembranes, with a special attention devoted to their effect on the mechano-elasticity/viscoelasticity of biomembranes. Low IL-doses have been defined as concentrations of ILs which are low enough to not cause any alteration to the overall biomembranes’ structural stability. In this context, we have shown by neutron reflectivity (Benedetto et al. 2014b) and classical molecular dynamics simulations (Benedetto et al. 2015) the insertion mechanism and the partitioning of IL-cations into phospholipid bilayers (Fig. 2). At low doses, (i) IL-cations insert into the lipid structure of phospholipids bilayers in a time scale of few nanoseconds; (ii) this insertion process is driven by the electrostatic attraction force between the positively charged imidazolium ring of the IL-cation with one of the electronegative oxygen atom in the lipid head, (iii) and it is stabilized by Van der Waals forces between the tail of the IL-cation and the tails of the phospholipid, which are both hydrophobic; (iv) the location of the IL-cation is always in the region between the heads and the tails of the phospholipids, and (v) it stays almost always in the outer leaflet region; (vi) the insertion process will reach a dynamical equilibrium with the amount of IL-cations in the order of 1-to-7 IL per 10 phospholipids; and (vii) it is not fully reversible. Furthermore, by AFM, we have shown that ILs dispersed at low doses at the bilayer-water interface are able to change the mechano-elasticity of phospholipid bilayers without affecting their overall structure and stability (Rotella et al. 2018), and that this holds also at cell level (Benedetto and Ballone 2018a) affecting cell migration and adhesion (Kumari et al. 2020). In this context, more specifically, we have shown that sub-toxic doses of imidazolium ILs are able to enhance cell migration in human breast cancer cell line MDA-MB-231 by reducing the elasticity and the penetration resistance of the cellular lipid membrane (Fig. 3), and that both cell migration and membrane elasticity can be tuned by IL-concentration and IL-chain length (Kumari et al. 2020).

These are just few examples of the still-growing biophysical and chemical-physical research efforts in the field. For an almost up-to-date overview of this research field, we propose to the interested reader two reviews that we have recently authored on this subject—one on the interaction between ILs and biomolecules (Benedetto and Ballone 2016) and the other one dedicated entirely to biomembranes (Benedetto 2017)—as well as a recent *Feature Article* highlighting the potential applications of ILs in bio-nanotechnology and bio-nanomedicine from a chemical-physical prospective (Benedetto and Ballone 2018a). Furthermore, a special issue entirely dedicated to ILs and biomolecules has been recently published (Benedetto and Galla 2018).

## Mechanisms of action of ILs

In what follows, we are presenting the state of the art of the MoAs of ILs towards living cells. In few cases, we will comment results obtained on model systems including lipid liposomes and supported lipid bilayers. We have organized and distributed the results of the relevant literature in subparagraphs organized by the relevant MoAs and inspired by the much more scientific literature published on the MoAs of antibiotics and drugs (Brogden 2005; Kohanski et al. 2010; Blair et al. 2015; Mookherjee et al. 2020). As you will see, some MoAs of ILs are very “populated,” whereas for others, there are very few examples reported in the literature so far.

### ILs and cellular membranes—part 1: “soft interactions”

**Fig. 4:**
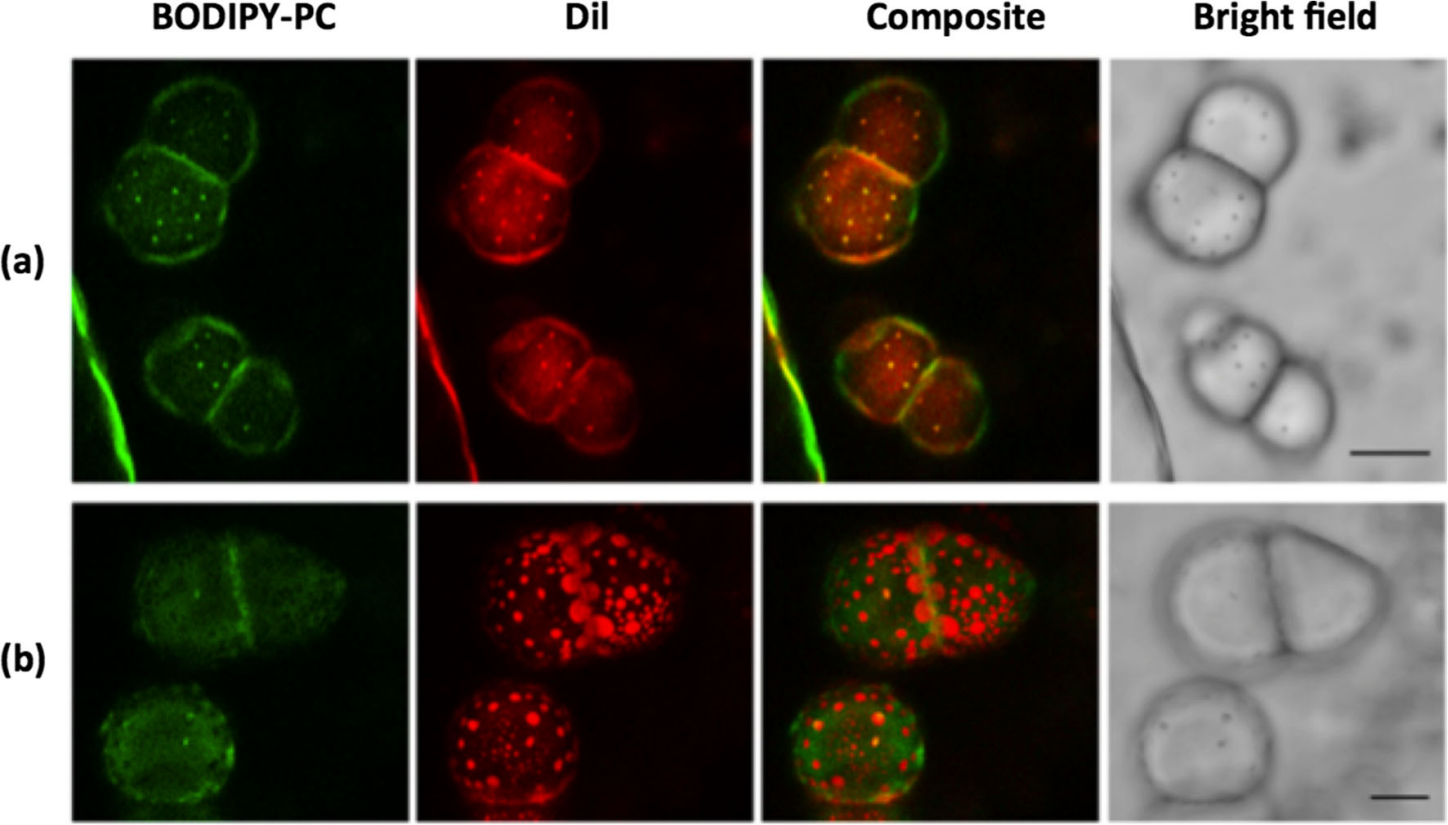
Bilayer domain fluidization of small bulged domains to flat large domains with enhanced dye specificity in the presence of 10% of the double-tail imidazolium-based IL [C_15_IMe][HI] from Drücker et al. (2017). Giant unilamellar vesicles of **a** DOPC/SSM/Chol (33:33:33) and **b** DOPC/SSM/Chol/[C_15_IMe][HI] (33:23:33:10) at 38 °C, scale 20 μm. Figure reproduced with permission from the publisher

ILs could diffuse into the cellular membrane and alter the phospholipids’ arrangement, the membrane potential, and the overall fluidity and viscoelasticity of the membrane. Changing the fluidity of the cell membrane could, for example, impact the diffusion rate and the overall stability of membrane proteins and, in turn, indirectly affect their biochemical function. This could impact several cell biochemical and biophysical processes, including recognition, transportation, signaling, migration, adhesion, division, and mechanotransduction, which could eventually lead to different effects up to cell death by both apoptosis and necrosis. In the specific case of lipid raft domains, the variation in the arrangement of lipids and in the overall fluidity, which could be induced by the absorption of ILs, could alter the ability of lipid raft domains to cluster specific membrane proteins needed to carry out specific biochemical functions as well as affect the distribution of glycolipids and glycoproteins involved in cell recognition. ILs could also affect the overall stability of lipid raft domains which could lead to cell death. It is interesting to mention here that (i) rearrangement of lipid domains by ILs has been observed on model systems (Drücker et al. 2017; Bornemann et al. 2020), see for example Fig. 4; (ii) insertion of antimicrobial peptides can induce delocalization of membrane proteins (Wenzel et al. 2014); (iii) alteration in membrane potential (and in the proton motive force) affect protein localization and cell division (Strahl and Hamoen 2010; Wilson et al. 2016); and (iv) antibiotics can inhibit the cell envelope synthesis by interfering with fluid membrane microdomains (Müller et al. 2016).

**Fig. 5:**
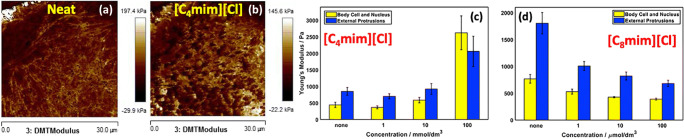
AFM scan of the local elastic modulus of osteoblast cells **a** before and **b** after the addition of [C_4_mim][Cl] at 0.1 M concentration, showing the overall reduction of the cell membrane elasticity and the destabilization of the cytoskeleton motif induced by the IL (Benedetto and Ballone 2018a). In **c** and **d**, Young’s modulus mean values ± errors for MDA-MB-231 cells interacting with different concentration of [C_4_mim][Cl] and [C_8_mim][Cl], respectively (Galluzzi et al. 2018). Figures reproduced with permission from the publisher

**Fig. 6:**
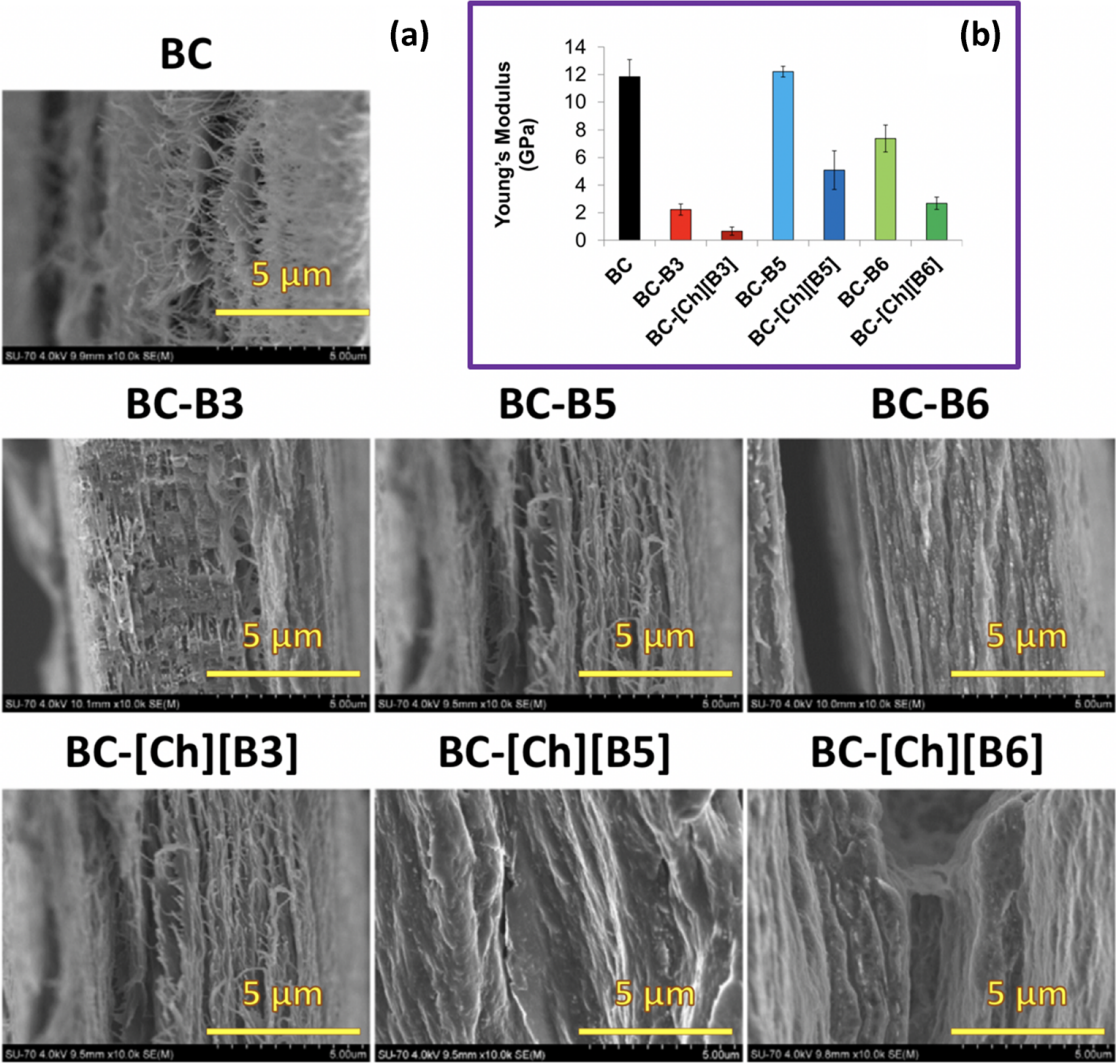
**a** SEM images (cross sections at 10k magnification) and **b** Young’s modulus of bacterial membranes (BC) from the bacterial strain *Gluconacetobacter sacchari* neat (BC, first row), treated with vitamins B (BC-Bx, second row) and treated with choline-based vitamin B ILs (BC-[Ch][Bx], third row). Adapted from Chantereau et al. (2020) and reproduced with permission from the publisher

Several of these MoAs of ILs have been observed, more specifically:

- Rounding up of cell border has been observed in human keratinocyte HaCaT cell line after incubation in [BMPY][TFSI] (Hwang et al. 2018).

- Blebs on cell surface of HeLa cells have been observed after incubation in [C_2_mim][BF_4_] (Wang et al. 2007).

- Membrane shrinkage and lipid peroxidation have been observed in human hepatocarcinoma QGY-7701 cells incubated in [C_8_mim][Cl] (Jing et al. 2013).
- Membrane blebbing, blurring, and shrinkage have been observed in the insect cell line *Spodoptera frugiperda* 9 (Sf-9) following incubation in [C_2_mim][Br] (Wu et al. 2018).

- Alterations in cell elasticity induced by ILs have been observed for the first time by Benedetto and Ballone (2018a) on osteoblast cells incubated in [C_4_mim][Cl] (Fig. 5). The same group has also shown that ILs can affect the mechano-elasticity of phospholipid bilayers (Rotella et al. 2018; Kumari et al. 2020).
- Variation in the Young’s modulus of MDA-MB-231 cells has been observed after incubation in [C_n_mim]-based ILs (Fig. 5). The reduction correlates positively with the length of the imidazolium IL-cation tail and has been associated to the ability of the ILs to alter the membrane-actin cytoskeleton wall (Galluzzi et al. 2018).

- A drop in the in-plane elasticity of the cellular lipid membrane has been measured in the human liver cancer cell line Huh7.5 incubated in [C_10_mim][BF_4_], and has been associated to the rearrangement of lipids due to the presence of the ILs in the lipid region. Interestingly, no alteration in either cell cycle or DNA damage has been observed at IL-concentrations around the EC50 value (Bhattacharya et al. 2018; Bakshi et al. 2020).
- Reduction of the Young’s modulus has been observed in *Gluconacetobacter sacchari* membranes incubated in cholinium-cation vitamin B-anion ILs (Chantereau et al. 2020), see Fig. 6.

- Loss of adhesion ability has been recorded in the case of PC12 cells incubated in [C_8_mim][Br] (Li et al. 2012a).

- Alteration in cell division has been observed in HeLa cells following incubation in ethyl-substituted imidazolium, pyridinium, and ammonium ILs; no such indication has been registered for longer chain-substituted ILs (Wang et al. 2007).

- Phosphatidylserine lipids have been observed in the outer cell membrane leaflet in human epidermoid carcinoma A431 cells incubated in [C_11_mim][Cl], [C_15_mim][Cl], and [C_17_mim][Cl] ILs. Usually, in healthy cells, this lipid is constrained into the cell membrane inner leaflet, because its exposure to the extracellular environment is a signal of cell apoptosis (Malhotra et al. 2014).

- Increase in the trans/cis ratio of unsaturated fatty acids has been observed in the soil bacterium *Pseudomonas putida* cell membrane after incubation in ammonium-based ILs (Piotrowska et al. 2017). It has been highlighted that these ILs directly affect bacterial membrane fluidity and activate membrane stress response.

- A correlation between surface activity of imidazolium-based ILs (i.e., surface tension reduction in aqueous solutions induced by ILs) and their toxicity activity against bacteria (including both Gram-positive and Gram-negative categories) and fungi has been recorded and linked to their MoA (Łuczak et al. 2010).
- The accumulation of [P_6,6,6,14_][NTf_2_] into the cell membrane of *Escherichia coli* (*E. coli*) cells has been measured by Fourier transform infrared (FT-IR) spectroscopy (Cornmell et al. 2008).

- Ex vivo lipid extraction in (porcine) skin by a choline-based IL has been observed and a theoretical model that elucidates how IL-induced skin structural changes result in faster macromolecular diffusion for enhanced permeability has been proposed (Tanner et al. 2018; Qi and Mitragotri 2019).

### ILs and cellular membranes—part 2: “hard interactions”

The interaction between ILs and cell membranes could lead to much stronger effects than the ones reported in the section above, for example, ILs could alter the membrane permeability, nucleate pores, and even disrupt the entire membrane. In these first two cases, ILs could indirectly perturb the biochemical gradients between the extracellular environment and the cytoplasm, and could also induce either the diffusion of the intracellular content outside the cell or the penetration of extracellular material into the cytoplasm. In all these cases, the biochemical function of the cellular membrane and several membrane proteins could be strongly affected, leading to cell death. Important to highlight here is that the permeabilization of the cellular membrane is also one of the most common MoAs of antibiotics, e.g., Mwangi et al. (2019).

**Fig. 7:**
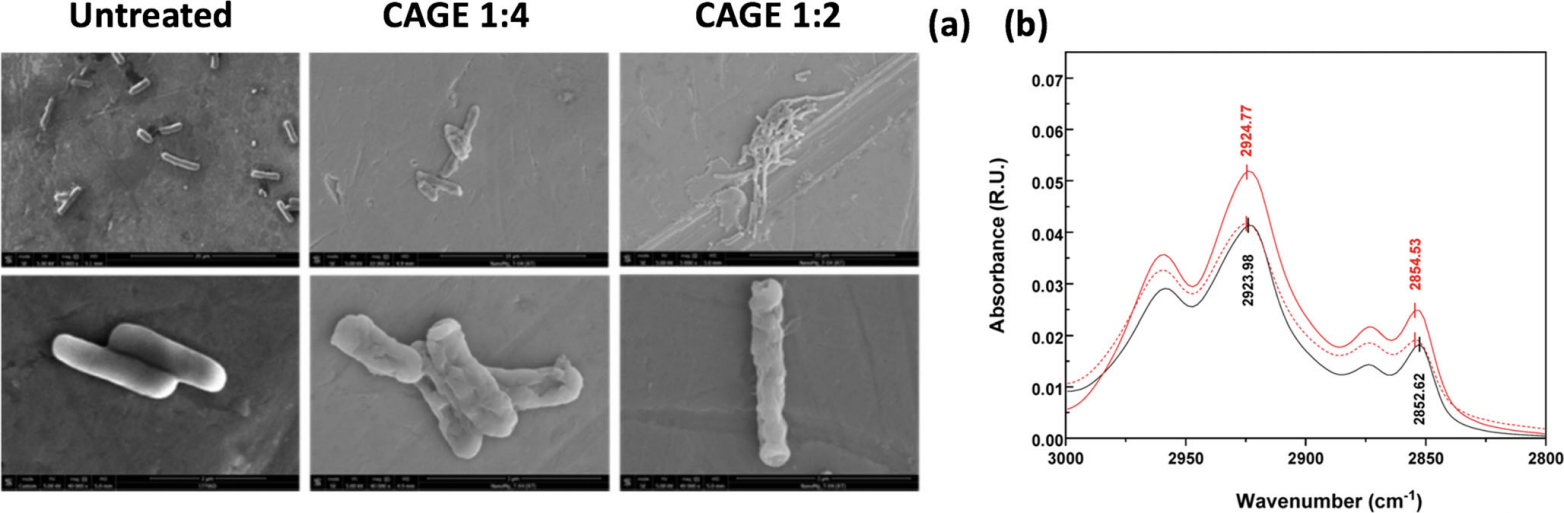
*E. coli* membrane disruption induced by choline- and geranate-based IL (CAGE) variants with varying ratios of choline and geranic acid. **a** SEM images of *E. coli* cells untreated and incubated in CAGE variants for 2 h at 37 °C, showing surface disruption on CAGE-treated cells. Magnification of top row images is × 5000−10,000 and bottom row images is × 40,000. **b** Fourier transform infrared spectroscopic analysis of treated cells show an altered lipid profile similar to phase transition, indicating the disruption of the lipid bilayer conformation. Cells incubated in sublethal doses of 1:2 CAGE for 2 h (dashed red line) or 24 h (solid red line) exhibited an increase in peak heights indicative of lipid content compared with an untreated control (solid black line). An increase in vibrational frequency of CH_2_ stretching was observed at 2853 and to a lesser extent at 2924 cm^−1^. Adapted from Ibsen et al. (2018) and reproduced with permission from the publisher

Several of these MoAs of ILs have been observed, more specifically:

- Alteration in membrane permeability has been detected in [C_8_mim][Cl]-treated PC12 cells (Li et al. 2012b).

- Enhanced membrane permeability has been observed in fish ovary CCO cells after incubation in [C_n_mim]-based ILs; [C_4_mim][NTf_2_] and [C_10_mim][NTf_2_] induced also membrane disintegration (Radošević et al. 2013).

- Reversible changes in membrane potential (which activates endogenous Ca^2+^ channels) and pore formation have been observed in primary cultures of dorsal root ganglion DRG neurons and human embryonic kidney HEK-293 cells incubated in 1,3-alkylpyridinium salts (McClelland et al. 2003; Scott et al. 2000). Interestingly, the presence of extracellular zinc seems to attenuate those effects. It has been suggested that the reversibility of the process could be used to deliver materials to the intracellular environment without cell damage. Several possible explanations have been given to explain the reversibility in pore formation, membrane potential, input resistance, and current, including the possibility that the larger poly-IL molecules may be sufficiently flexible that lipids can rearrange themselves after pore formation and thus block the ion-conducting pathways through the cell membrane.

- Membrane damage has been observed in red blood cells (RBC) incubated in [C_4_mim][Br], [C_10_mim][BF_4_], and [BTDA][Cl] (Thamke et al. 2017).

- Destabilization of the plasma cellular membrane of human corneal epithelial HCE cells and hemolysis of RBCs have been observed after incubation in [P_14444_][Cl] and [P_14444_][OAc] (Ruokonen et al. 2018). In the same study, it has been shown that these two ILs switch the zeta potential of model lipid liposomes from negative to positive, evidencing a strong and possibly irreversible sorption of the ILs into the liposome. To be noted is that the concentrations at which the zeta potentials turned positive are relatively close to the EC50 values of these ILs.

- Cellular membrane destabilization has been observed in mammalian murine fibroblast cells NIH/3T3 incubated in the [C_8_quin][Br] and [C_14_quin][Br] alkyl-quinolinium bromide ILs (McLaughlin et al. 2011).

- Destabilization of the cell membrane has been observed in PC12 cells incubated in oxygenated and alkyl imidazolium ILs; [C_4_mim][BF_4_] resulted to be the most toxic compounds (Samorì et al. 2010).

- Membrane damage in PC12 cells has been observed after incubation in [C_8_mim][Br] (Li et al. 2012a).

- Loss of membrane integrity has been registered in human breast cancer MCF-7 cells incubated in [MPPyrro][Br] (Kumar et al. 2009).

- Membrane destruction by [C_2_mim][BF_4_] has been observed on HeLa cells (Wang et al. 2007).

- Membrane damage by ILs seems also to be a common MoA of ILs against bacteria. For example, membrane disruption has been observed in *E. coli* incubated in choline- and geranate-based ILs (Ibsen et al. 2018), see Fig. 7.

- Membrane damage has been observed in both Gram-negative and Gram-positive bacteria (i.e., *E. coli* and *Staphylococcus aureus*) incubated in piperazinium- and guanidinium-based ILs (Yu et al. 2016a), see Fig. 8. Interestingly, *E. coli* appears to be more susceptible to be destroyed by ILs than *S. aureus*, pointing to different interactions of ILs with bacterial membrane and cell wall, which are different between gram-negative and gram-positive bacteria.

- Membrane damage has been observed in *Clostridium* sp. incubated in [EtPy][BF_4_] and [EtPy][CF_3_COO] ILs (Zhang et al. 2014).
- Increase in membrane permeability was observed in *E. coli* DH5a incubated in [C_8_mim][Cl] (Jing et al. 2014).

- Permeabilization of the cell membrane has been observed in bacteria (*E. coli*), yeast (*Saccharomyces cerevisiae*), and RBCs after incubation in [C_8_mim][Cl] (Cook et al. 2019), see Fig. 9. In the same study, it has also been shown that [C_8_mim][Cl] is able to induce permeability in large unilamellar vesicles of DOPC/DOPG at its CMC.

- Increase in membrane permeability and membrane damage has been also observed in cells extracted from algae, *Chlorella vulgaris* (*C. vulgaris*) and *Scenedesmus quadricauda* (*S. quadricauda*), after incubation in [C_n_mim][Cl] ILs (Deng et al. 2020).

- Membrane damage has been observed in marine macroalga *Ulva lactuca* (*U. lactuca*) incubated in [C_12_mim][Br] (Kumar et al. 2011).

- Membrane and cell wall damage by ILs has also been observed in fungi. For example, it has been shown that the phosphonium-based ILs [P_444n_][Cl] can induce membrane damage in conidia of the filamentous fungus *Aspergillus nidulans* (Petkovic et al. 2012).

- Morphological membrane damages have also been observed in *Caenorhabditis elegans*, a well-studied free-living soil roundworm with a transparent anatomy, after incubation in [C_n_mim][Cl] ILs (Swatloski et al. 2004).

- Rupture of the membrane and increased release of cytoplasmic contents have been observed in Sf-9 cells following incubation in [C_2_mim][Br] (Wu et al. 2018).

**Fig. 8:**
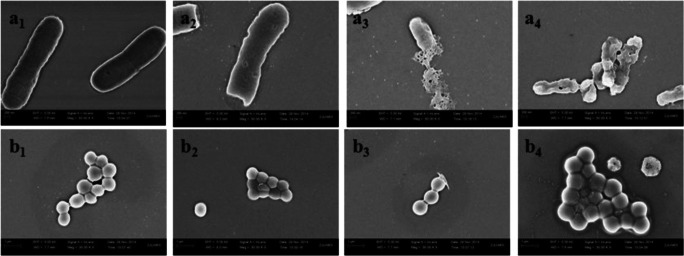
SEM images of **a** *E. coli* and **b** *S. aureus* bacteria treated with [C_2_pi][BF_4_] for 12 h. (a_1_, b_1_) control, untreated; (a_2_, b_2_) treated with 0.1 mg mL^−1^; (a_3_, b_3_) treated with 1.0 mg mL^−1^; (a_4_, b_4_) treated with 2.0 mg mL^−1^. Figure taken from Yu et al. (2016a) and reproduced with permission from the publisher

**Fig. 9:**
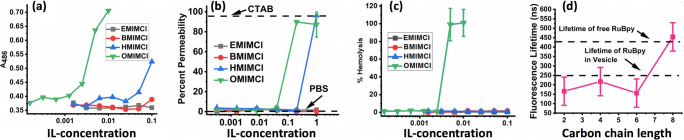
Permeabilization induced by [C_n_mim][Cl] ILs on bacteria (*E. coli*), fungi (*S. cerevisiae*), ovine RBCs, and large unilamellar 98%DOPC/2%DOPG vesicles (LUV). **a** *E. coli* outer membrane permeabilization by varying IL-concentrations. Data represent absorbance measured after 30 min of exposure to ILs; all data are averages of 3 replicates. **b** Effect of IL-chain length on *S. cerevisiae* permeability. Cells with positive fluorescence at 575 nm were considered permeable. Exposure to cetyltrimethylammonium bromide (CTAB) (102 μM) yielded 94% permeable cells vs. PBS treatment 4% (dotted lines). **c** Ovine RBC permeabilization by varying IL-concentrations. Data represents hemolysis induced by 1 h exposure to ILs. Percent hemolysis was calculated by normalizing against cells treated with Triton X-100 set to 100% leakage. All data are averages of 3 replicates. **d** Laser-induced fluorescence lifetimes of LUVs entrapped with Ru(bpy)_3_^2+^ in the presence of 0.5 M ILs. Adapted from Cook et al. (2019) and reproduced with permission from the publisher

### ILs and membrane proteins

ILs could directly interact with and bind to membrane proteins and enzymes affecting their biochemical function. For example, ILs could bind to transmembrane lipid-transporter proteins (e.g., flippases and floppases) responsible for aiding the movement of phospholipid molecules between the two leaflets of the cell membrane, and to maintain their asymmetric lipid composition. ILs could also bind to transmembrane protein channels (e.g., porins and aquaporins) responsible for the passage of water, other (ionic) molecules, and nutrients. Similar MoAs have been observed for antibiotics (Feng et al. 2015). Moreover, ILs could use both lipid-transporter proteins and transmembrane protein channels to diffuse into the cytoplasm. ILs could bind to glycoproteins and glycolipids important in the recognition of the cell by the immune system, or to the several proteins responsible for cell adhesion and mobility. ILs could also affect the overall viscoelasticity and stability of cells and of cell colonies by binding to cytoskeleton-anchored transmembrane proteins as well as to cellular-cellular channels responsible for the exchange of molecules between (adjacent) cells (or by acting on the extracellular matrix). ILs could finally interfere with several signaling pathways occurring at the cellular membrane level. For example, in bacteria, ILs could bind or destabilize the peptidoglycan structure, which could affect the ability of the relevant enzyme to synthesize it, or in a complementary way, they could alter the function of the enzymes responsible to the synthesis of the cell wall (e.g., peptidoglycan). Similar MoAs have been observed for antibiotics (Ling et al. 2015; Batson et al. 2017; Grein et al. 2020). More in general, ILs could interfere with active transporters also by affecting the conversion from adenosine diphosphate (ADP) to adenosine triphosphate (ATP), for example, by directly binding ADP/ATP. ILs could also affect electron and proton transfer pathways.

**Fig. 10:**
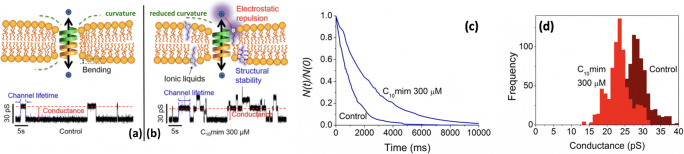
Cartoon representation of the effect of ILs on the structure and ionic conductivity of gramicidin A embedded in a supported DPhPC/n-decane phospholipid bilayer. Each current trace represents events **a** prior to and **b** after addition of 300 μM [C_10_mim][Cl] (applied voltage 200 mV). In **c**, lifetime distribution of gramicidin A dimer: *N*(*t*) is the number of channels with lifetimes longer than time *t*. In **d**, conductance transition amplitude histograms depending on presence of IL. Adapted from Ryu et al. (2015) and reproduced with permission from the publisher

Several of these MoAs of ILs have been observed, more specifically:

- Inhibition of the enzyme acetylcholinesterase (AChE) in PC12 cells has been observed after incubation in [C_4_mim][BF_4_] (Samorì et al. 2010). In this cell line, four AChE monomers are organized in a globular tetramer directed onto the membrane surface for its cholinergic function by a membrane anchor protein.

- Inhibition of p-glycoprotein, which is a plasma membrane protein exporting drugs out of the cell, has been observed in several human cancer cell lines originating from breast and colon cancers after incubation in a series of newly synthesized imidazolium ILs tethered benzothiazole moieties with fluorinated counter anions (Al-blewi et al. 2019).

- Inhibition of the Na-K-ATPase, an enzyme located onto the cell membrane and responsible for cell growth by regulating the in and out transport of, respectively, K^+^ and Na^+^ ions has been observed in CCO cells incubated in [C_n_mim][NTf_2_] ILs (Radošević et al. 2013; Stolte et al. 2006). It has been suggested that the inhibition is due to the IL anion ability to form free fluoride ions.
- The binding of phosphoinositide 3-kinases (PI3Ks) p110α domain with a series of di-cationic pyridinium-based ILs have been tested by in silico molecular docking approach to investigate the potential MoA of these ILs that show cytotoxicity in the millimolar range against human breast cancer (MCF-7, MDA-MB-231, and T47D) and colon cancer (Caco-2) cell lines. The docking study revealed that this family of ILs exhibits bonds with one or more amino acids in the receptor active pocket site of the enzyme, and that the IL exhibiting the highest cytotoxicity has the highest binding energy (Rezki et al. 2018). PI3Ks belongs to the family of lipid kinase enzymes, it regulates cellular growth and apoptosis in cancer cells, and catalyzes the phosphorylation of phosphatidylinositol that can activate the serine/threonine kinase AKT, which regulates several signaling pathways controlling cell survival, proliferation, apoptosis, and motility.

- The lifetimes of gramicidin A channels have increased by incubation in [C_n_mim][Cl] ILs, which have been suggested to stabilize the compressed structure of the lipid bilayer (Ryu et al. 2015), see Fig. 10. Decrease rate of ion flux through the channels has also been observed in the same study and associated with the ability of the ILs to change the membrane surface charge. To be noted, both IL-effects have shown to increase with increasing concentration and alkyl chain length.
- Diffusion through biological nanopores, such as OmpF and its mutant D113A, has been observed for [C_4_mim][Cl] (Modi et al. 2011).

- Diffusion into the cytoplasm via special membrane transporter proteins (e.g., PEPT1 transporter), similar to some amino acid prodrugs, has been suggested as a MoA of amino acid–based ILs, showing a much higher toxicity against NIH/3T3 cells in comparison with the equivalent most common imidazolium ILs (Egorova et al. 2015).

- Interaction between ILs and membrane proteins and enzymes has also been observed and proposed as a MoA of ILs against bacteria. For example, it has been suggested that in *S. aureus* bacteria incubated in piperazinium [BF_4_]– and guanidinium [BF_4_]–based ILs, the free fluorine resulting from the hydrolysis of the F-containing group can inhibit the Na-K-ATPase, which by transporting K^+^ into the cell and Na^+^ outwards regulate the cell growth (Yu et al. 2016a).

As a result of their interactions at cell membrane level, ILs could reach the intracellular environment by several routes. More specifically, once diffused into the outer cellular membrane, they could flip and then diffuse into the cytoplasm. They could also reach the intracellular region via transmembrane lipid-transporter proteins (e.g., flippases and floppases), transmembrane protein channels (e.g., porins and aquaporins), and, of course, by self-induced cell permeability and pores. Once in the cytoplasm, ILs could interact with several biomolecules and protein complexes (firstly driven by Coulombic attraction), and with organelles (driven also by the lipophilicity of ILs); reach the cell nuclei; and also interfere with several signaling pathways including the ones responsible for cell cycle, cell growth, cell proliferation and differentiation, cell mobility, and apoptosis. The cytoplasm is a very crowded environment which comprises a huge variety of biopolymers that are predominantly anionic (e.g., DNA, ribosomes, RNA, and most proteins). The presence of ILs (cations) could alter the electrostatic equilibrium of the cytoplasm and change its viscosity, affecting the rate of diffusion of the biopolymers, as shown for antimicrobial peptides (Zhu et al. 2019). In this respect, it could be interesting to investigate how ILs affect protein mobility in cells and bacteria by tracking single molecules, e.g., Kapanidis et al. (2018).

### ILs, cytoplasmatic proteins and enzymes, RNA, and ribosomes

ILs could interfere with protein synthesis by binding to RNA that is one of the most charged molecules in the cytoplasm, or to ribosomes which are the machineries that synthesize proteins; RNA contains the genetic code needed by the ribosomes for protein synthesis. This can lead to either defects (mutations), upregulations, or downregulations in the expression of proteins, or to the overall inhibition of the ability of ribosomes to protein synthesis. Alteration in protein synthesis can affect the cell cycle, cell growth, and cell differentiation, and can lead to cell death. These types of MoAs have been already observed for antibiotics, e.g., Holm et al. (2016) and Marks et al. (2016). Direct affinity and interaction between ILs and nucleic acids (DNA and RNA), on the other hand, has been observed and it is at the base of several applications in bio-nanotechnology, e.g., magnetic ILs have been used for RNA extraction (Zhu et al. 2020). Moreover, ILs have been shown to interact with purified/isolated proteins and enzymes, so they could directly interact with the proteins and enzymes present in the cytoplasm. For example, they could bind cell cycle regulatory proteins or activate or inactivate proteins responsible for cell growth, cell replication, and apoptotic pathways.

**Fig. 11:**
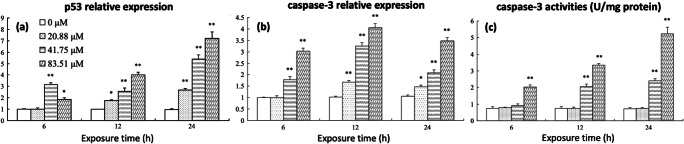
The transcriptional levels of **a** p53 and **b** caspase-3, and the enzymatic activity of **c** caspase-3 in HepG2 cells incubated in [C_8_mim][Br]. Adapted from Li et al. (2015) and reproduced with permission from the publisher

**Fig. 12:**
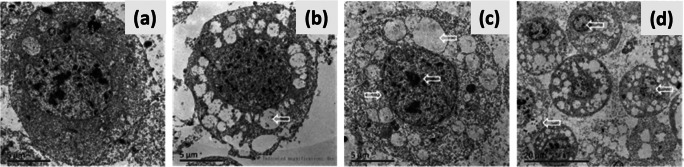
Representative transmission electron microscope (TEM) images of Sf-9 cells **a** untreated and incubated in 0.6250 mg/mL [C_2_mim][Br] for **b** 12 h, **c** 24 h, and **d** 48 h. The arrows indicate the formation of vesicles in the cytoplasm induced by the presence of the IL. Adapted from Wu et al. (2018) and reproduced with permission from the publisher

Several of these MoAs of ILs have been observed, more specifically:

- Upregulation of cytochrome P450 members including CYP1A1, CYP2E1, and CYP3A, at mRNA level, has been observed in mouse mammary carcinoma EMT6 cells incubated in [C_8_mim][Cl]; this suggested that imidazolium-based ILs can activate CYPs (Li et al. 2013).

- Activation of caspase-3 has been observed in PC12 cells following incubation in [C_8_mim][Br] (Li et al. 2012a). Caspase-3 is activated in the apoptotic cell both by extrinsic (programmed death ligand, PD-L1) and intrinsic (mitochondrial) pathways.

- The transcriptions of caspase-3, caspase-8, caspase-9, p53, and Bax turned out to be markedly upregulated, while Bcl-2 transcription significantly downregulated in HepG2 cells incubated in [C_8_mim][Br] (Li et al. 2015), see Fig. 11. Caspases are a family of cysteine proteases that are broadly grouped into initiators such as caspase-8 and caspase-9 and executioners such as caspase-3 and caspase-7 of apoptosis, which play a key role in apoptosis induction and execution. Particularly, caspase-8 is a key initiator in the death receptor-mediated pathway, while caspase-9 plays a critical role in mitochondria-mediated pathway. The activated caspase-8 or caspase-9 acts directly on downstream executioner caspase-3 which is responsible for the cleavage of death substrates and execution of apoptosis. p53 is an important anti-oncogene that plays a key role in apoptosis of cancer cells; moreover, Bcl-2 and Bax families suppress and promote apoptosis, respectively, and their proportion in cells can decide death (by apoptosis) or survival of the cell.

- Activation of the effector caspase-3 and caspase-7 by [C_15_mim][Cl] and [C_17_mim][Cl], and activation of the initiator caspase 8 by [C_11_mim][Cl] and [C_17_mim][Cl] has been observed in A431 cells (Malhotra et al. 2014). Taken together, these sets of data suggested that these ILs can activate an extrinsic apoptotic pathway.
- Upregulation of the transcriptional levels of p53 and Bax and downregulation for Bcl-2 have been observed in HepG2 cells incubated in [C_16_mim][Cl] (Wan et al. 2018).

- Inhibition of superoxide dismutase (SOD) and catalase (CAT) activities, reduction of glutathione (GSH) content, and increase of the activity of caspase-3 have been observed in QGY-7701 cells incubated in [C_8_mim][Cl] (Jing et al. 2013).

- Selective inhibition of butrylcholinesterase enzyme has been observed in HeLa, H460 (lung), and NIH/3T3 cell lines after incubation in imidazolium aurate-based ILs (Sioriki et al. 2019). The selectivity character of these ILs has been determined by investigating their inhibition ability against several enzymes, including prolyl-endopeptidase, butryl-cholinesterase, tyrosinase, dipeptidyl peptidase IV, and carbonic anhydrase.

- Inhibition of the activity of the acetylcholinesterase enzyme has been observed in IPC-81 rat promyelocytic leukemia cell lines incubated in 1-alkoxymethyl-3-hydroxypyridinium cations + acesulfamate anion ILs (Stasiewicz et al. 2008).

- Increase in enzymes activity and proteins damage has also been observed in the plant *Vicia faba (V. faba*) due to incubation in imidazolium-based ILs (Xu et al. 2020; Liu et al. 2015a; Liu et al. 2016).

- Modulation of three relevant intracellular signaling pathways, i.e., MEK, NF-kB, and JNK, by the novel betulinic acid (BA)–derived IL [P_6,6,6,14_][BA] has been observed in HFF-1 (fibroblast), HepG2, T47D, and A459 cell lines. More specifically, the addition of this IL downregulates NF-kB and upregulates JNK in HFF-1 cells, and upregulate NF-kB in HepG2 and T47D cells (Silva et al. 2019).
- Formation of vesicles in the cytoplasm of HeLa cells after incubation in [C_2_mim][BF_4_] has also been observed (Wang et al. 2007).
- Formation of vesicles in the cytoplasm has also been observed in Sf-9 cells following incubation in [C_2_mim][Br], leading to organelle breakdown (Wu et al. 2018), see Fig. 12.

### ILs, mitochondria, and ATP

ILs could diffuse into the mitochondria (membrane) and interfere with the function of this very important organelle including the generation of ATP, and with several signaling processes involved in cell cycle, cell growth, cell differentiation, and cell death. For example, the mitochondrial membrane potential, which is essential to generate ATP in mitochondria, can collapse if mitochondrial permeability transition pores open, which can also lead to the release of cytochrome C into the cytosol, a key event in the initiation of apoptosis. Moreover, alteration in the mitochondrial function can lead to the production of reactive oxygen species (ROS), and to lipid peroxidation, this can be triggered, for example, by increasing the protein expression levels of intercellular antioxidant enzymes. High ROS values could be induced by ILs either by alteration of the mitochondria function itself or by interfering with other processes in the cytoplasm, and they can damage many types of biological macromolecules, such as membrane lipids, DNA, and enzymes.

ILs could also directly interact with ATP, which is one of the most charged molecules present in the cytoplasm. ATP is the source of chemical energy responsible for the majority of cell processes, and the conversion from ATP to ADP is essential for the life of cells. ILs could either bind directly to ATP or affect the conversion from ATP to ADP. In both cases, the major cell processes could be affected, and this could lead to cell death. In this respect, there is only one study somehow (partially) related to this MoA of ILs in which very low level of ATP has been observed in PC12 cells treated with [C_8_mim][Cl] (Li et al. 2012b). Low level of ATP, however, can be also associated to mitochondrial dysfunction induced by ILs, apart from inactivation of ATP directly by ILs.

**Fig. 13:**
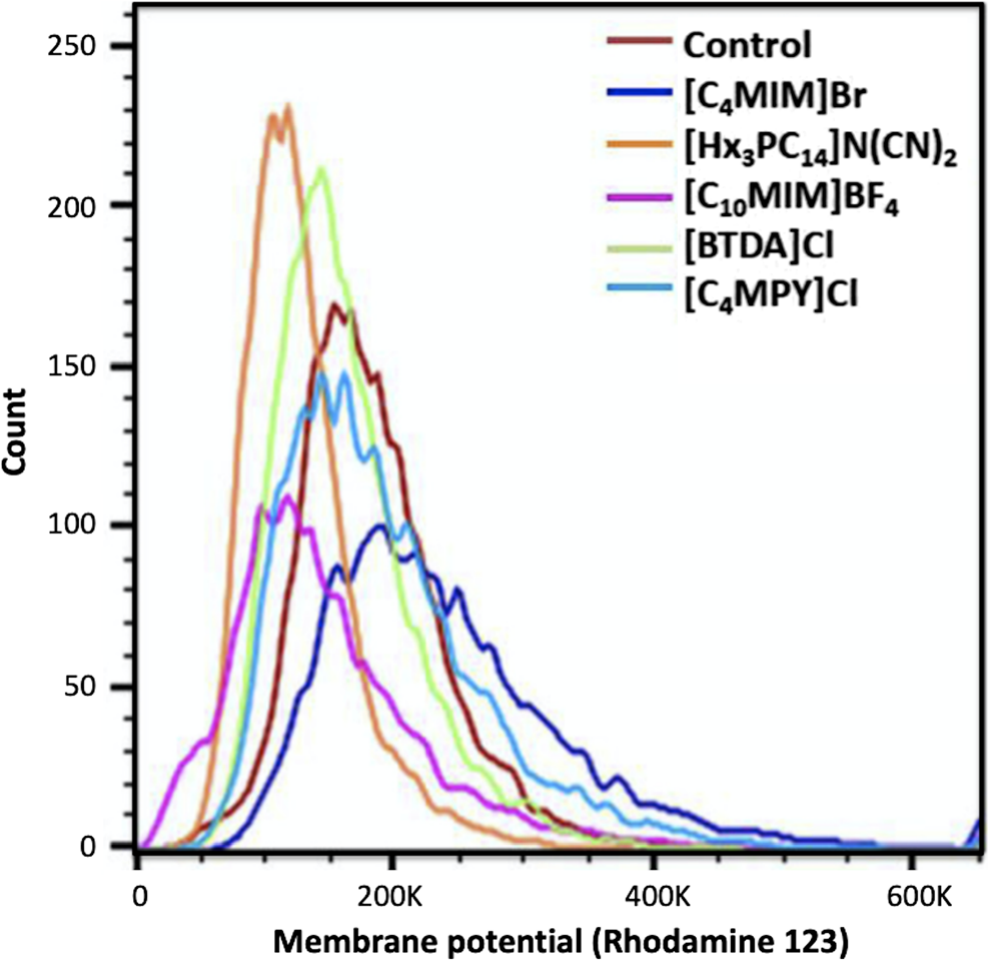
Shift in the mitochondrial membrane potential of MDA-MB-231 cells treated with various ILs. Figure taken from Thamke et al. (2019) and reproduced with permission from the publisher

**Fig. 14:**
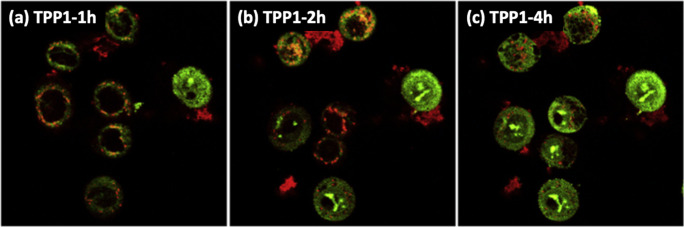
Representative pictures from live cell imaging of JC-1 stained RT112 cells **a** 1 h, **b** 2 h, and **c** 4 h after a treatment with 200 mM TPP1 IL. JC-1 is a mitochondrial dye that stains polarized mitochondria red and depolarized mitochondria green, and that reversibly changes color from red to green as the membrane potential decreases. After 1 h of incubation in TPP1, cells had red punctate markings which indicated the presence of intact (polarized) mitochondria, but as time passes, the shift from the red to green color indicates the loss of membrane potential.

**Fig. 15:**
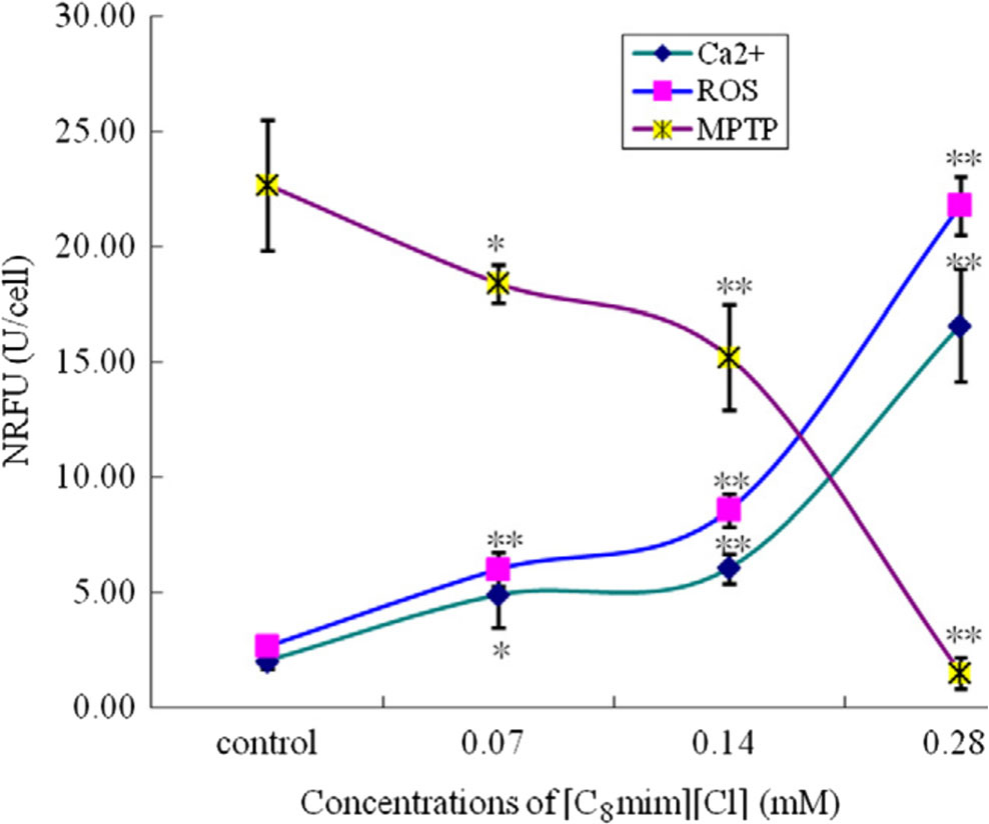
The intracellular Ca^2+^ contents, ROS levels, and opening of mitochondrial permeability transition pores (MPTPs) of PC12 cells after 24 h of incubation in [C_8_mim][Cl]. The results are presented as the normalized relative fluorescence units (NRFU) (U/cell). In MPTP detection, the decreased NRFU indicate that the IL may induce mitochondrial permeability transition in PC12 cells. Data is expressed as the means ± errors from three independent experiments with triplicate. Asterisk denotes a response that is significantly different from the control (*p < 0.05, **p < 0.01). Figure taken from Li et al. (2012a) and reproduced with permission from the publisher

Several of these MoAs of ILs have been observed, more specifically:

- Alterations in mitochondrial membrane potential and permeabilization have been reported in A431 cells incubated in [C_15_mim][Cl] ILs (Malhotra et al. 2014).

- Decrease in mitochondrial membrane potential has been observed in MDA-MB-231 cells incubated in [C_4_mim][Br] and [C_10_mim][BF_4_] (Thamke et al. 2019), see Fig. 13.
- Decrease in mitochondrial membrane potential has been observed in RT112 cells incubated in a new imidazolium IL with a triphenylphosphonium substituent, TPP1 (Stromyer et al. 2020), see Fig. 14. The same study shows that this mitochondrial membrane potential dysfunction triggers the release of cytochrome C that initiates cell apoptosis.

- Decrease in mitochondrial membrane potential has been observed in HeLa cells after incubation in [C_2_mim][BF_4_] (Wang et al. 2007). The same study reports that the production of ROS also increased as well as the concentration of intracellular calcium, suggesting that the cells undergo apoptosis in the presence of this IL.

- Mitochondrial dysfunction and overproduction of ROS has been observed in PC12 cells incubated in [C_8_mim][Br] (Li et al. 2012a) and in [C_8_mim][Cl] (Li et al. 2012b). Moreover, it has been proposed that [C_8_mim][Cl] is able to open the mitochondrial permeability transition pores inducing the release of mitochondrial Ca^2+^ into the cytoplasm, the excess of ROS, and the decrease of ATP, resulting eventually in cell apoptosis (Li et al. 2012b), see Fig. 15.

- Overproduction of ROS and lipid peroxidation has been observed in QGY-7701 cells incubated in [C_8_mim][Cl], suggesting that ROS-mediated oxidative stress may be responsible for cell apoptosis (Jing et al. 2013).

- Overproduction of ROS and decrease in glutathione content, an endogenous antioxidant, have been observed in HaCaT cells after incubation in [BMPY][TFSI] (Hwang et al. 2018).

- Overproduction of ROS, inhibition of superoxide dismutase and catalase, reduction of glutathione content, and increase in the cellular malondialdehyde level have been observed in HepG2 cells incubated in [C_8_mim][Br] (Li et al. 2015).
- Oxidative stress has been observed in HepG2 cells incubated in [C_16_mim][Cl] (Wan et al. 2018). More specifically, inhibition of superoxide dismutase, decrease in glutathione content, and increase in cellular malondialdehyde levels have been recorded.

- Generation of ROS and other effects, as oxidative damage and lipid peroxidation, have also been observed in bacteria, algae, and plants. It is interesting to comment here that ILs have been shown phytotoxicity as well (Pawłowska et al. 2019). The MoAs behind the IL phytotoxicity have been investigated and similar results to the case of human cell lines have been obtained. For example, in the plant *V. faba*, it has been observed that imidazolium-based ILs lead to the generation of ROS and then cause oxidative damage, including lipid peroxidation, protein damage, and DNA damage, which trigger an increase in antioxidant content and enzyme activity (Xu et al. 2020; Liu et al. 2015b; Liu et al. 2016).

- In the case of *Vibrio fischeri* bacteria, it has been shown that [C_2_mim]-based ILs are able to affect the content and the activity of triphosphopyridine nucleotide (NADPH), nicotinamide adenine dinucleotide (NADH), flavin mononucleotide, ATP, ROS, super-oxide dismutase, catalase, glutathione, and lipid peroxidases (Yu et al. 2016b).

- Generation of ROS and lipid peroxidation have been observed in the marine macroalga *U. lactuca* incubated in [C_12_mim][Br] IL (Kumar et al. 2011). These effects have been attributed to lipoxygenase (LOX) activity coupled with the induction of two LOX isoforms. Interestingly, pretreatment of the algal thallus with enzyme inhibitors such as diphenylene iodonium, sodium azide, cantharidin, and oxadiazoloquinoxalin-1-one, prior to [C_12_mim][Br] exposure, showed the regulation of ROS by the activation of membrane-bound NADPH-oxidase and cytochrome oxidase. Furthermore, an enhancement in several antioxidant enzyme activities (e.g., superoxide dismutase (SOD), ascorbate peroxidase (APX), and glutathione reductase (GR)), a reduction in glutathione peroxidase (GSH-Px) activity and a significant decrease in total chlorophyll content have been also registered. The IL exposure resulted in the accumulation of n-3 and n-6 fatty acids, indicating the induction of desaturase enzymes.

### ILs, chloroplast, and chlorophyll

In algae and plants, chlorophyll (Chl) content is a good indicator of the physiological state of cells, as it provides information on metabolite absorption and distribution, cellular energy use in preparation for photosynthesis, and can be associated with structural damage in chloroplasts. ILs could diffuse into and interact directly with chloroplasts and/or affect the photosynthesis by altering the charge transfer processes.

**Fig. 16:**
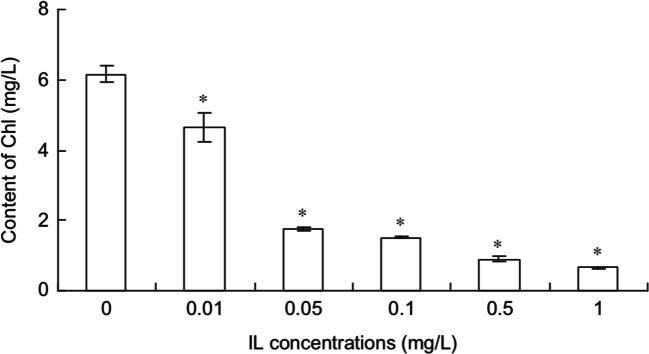
Effect of [C_10_mim][Br] on the content of Chl of *S. obliquus*. At the lowest IL-concentration of 0.01 mg/L (10^−1^ of EC50), a significant decrease in total Chl content, compared with the base value, is observed. Total Chl content continues to decrease with increase in IL-concentrations. Values are means ± errors of four replicates per treatment. Symbols were used to indicate significant differences when one-way ANOVA analysis for repeated measures was significant, *p < 0.05, as compared with control values. Figure was taken from Ma et al. (2010) and reproduced with permission from the publisher

**Fig. 17:**
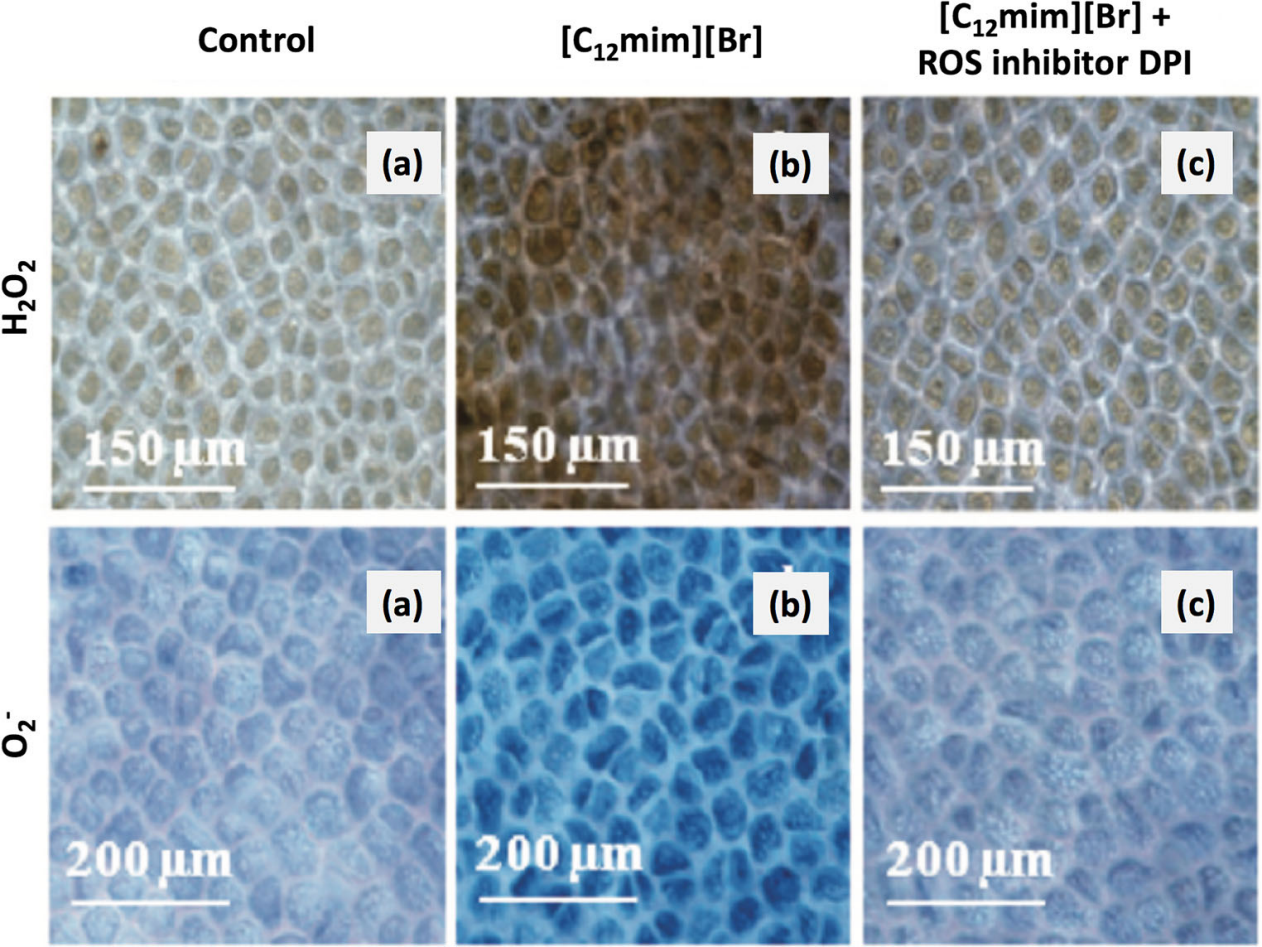
ROS generation (first row: H_2_O_2_, and second row: O_2_^-^) in *U. lactuca* **a** control, **b** exposure to [C_12_mim][Br] for 4 days, and **c** effect of pretreatment by the NADPH-oxidase inhibitor diphenylene iodonium (DPI). Adapted from Kumar et al. (2011) and reproduced with permission from the publisher

Several of these MoAs of ILs have been observed, more specifically:

- Inhibition and enhancement of Chl a have been observed in *S. quadricauda* and *C. vulgaris*, respectively, after incubation in [C_n_mim][Cl] ILs. The reduction in Chl content in *S. quadricauda* has been associated to the inhibition of the electron flow on the donor side of the photosystem II (PSII) reaction center, whereas its increase in *C. vulgaris* has been explained by an inhibition of the electron flow on the acceptor side of the PSII reaction center (Deng et al. 2020).
- A decrease in Chl total content has been observed in *Chlorella ellipsoidea* after incubation in [C_n_mim][Br] ILs (Ma et al. 2010), see Fig. 16.

- A significant decrease in total Chl content has been observed in the marine macroalga *U. lactuca* incubated in [C_12_mim][Br] IL (Kumar et al. 2011), see Fig. 17.

- A decrease in Chl total content has been observed in *Hordeum vulgare* after incubation in [C_n_mim][Br] ILs (Cvjetko Bubalo et al. 2014). Moreover, when seedlings were exposed to higher concentrations of ILs, the antioxidant system could not effectively remove ROS, leading to lipid peroxidation and damage of the photosynthetic system.

- Photosynthetic activity inhibition of *Pseudokirchneriella subcapitata* has been observed at relatively low concentrations (from 10 mM to 0.1 M) of imidazolium- and pyridinium-derived ILs (Pham et al. 2008).

- Reduction in total content of Chl a, Chl b, and carotenoids has been observed in the plant *V. faba* after incubation in imidazolium-based ILs (Liu et al. 2015a; Liu et al. 2016).
- Reduction in total content of Chl a, Chl b, and carotenoids has been observed in wheat seedlings after incubation in [C_8_mim][PF_6_] (Liu et al. 2014).
- Reduction in total content of Chl a and Chl b, damage of the PSII, inhibition of the transmission of excitation energy, and decrease in the efficiency of photosynthesis have been observed in green alga *Scenedesmus obliquus* (*S. obliquus*) exposed to 1-alkyl-3-methyl imidazolium tartrate IL (Liu et al. 2015b).
- Alterations in cell morphology have been observed in green alga *S. obliquus* incubated in [C_n_mim][Cl]: the cell wall of treated algae became wavy and separated from the cell membrane, chloroplast grana lamellae became obscure and loose, osmiophilic material was deposited in the chloroplast, and mitochondria and their cristae swelled (Liu et al. 2015c).
- Chl fluorescence parameters such as the maximum effective quantum yield of PSII (*F*_v_/*F*_m_), the potential activity of PSII (*F*_v_/*F*_0_), the yield of photochemical quantum (Y(II)), the photochemical quenching coefficient (qP), the non-photochemical quenching coefficient (NPQ), and the relative electron transport ratio (rETR) turned out to be affected in rice seedlings incubated in [C_n_mim][Cl], showing that these ILs can damage the PSII (Liu et al. 2015d).
- Chl fluorescence parameters (*F*_0_, *F*_v_/*F*_m_, *F*_v_/*F*_0_, Y(II), and NPQ) were affected in green alga *S. obliquus* after incubation in 1-decylpyridinium bromide IL, indicating a damage induced on PSII (Liu et al. 2018). Moreover, transfer of excitation energy was inhibited, photosynthetic efficiency was reduced, Chl content decreased, cell membrane permeability increased, and ROS level increased.

### ILs, DNA, and plasmids

DNA is one of the most charged intracellular molecules. In eukaryotic cells, for the majority of the cell cycle, DNA is stored in the cell nucleus; however, during mitosis, DNA is released in the cytoplasm. In prokaryotic cells, instead, DNA is dispersed in the cytoplasm for the whole duration of the cell life/cycle. ILs could bind directly to DNA in the cytoplasm or, after having diffused into the cell nuclei. DNA contains all the genetic code of the cell, so altering DNA (e.g., decrease DNA replication or induce DNA fragmentation) could lead to several effects including cell death and alteration of cell genome (which, however, is also at the base of gene therapy with ILs). Finally, we would like to mention that ILs could also interact with plasmids, which are dispersed in the cytoplasm and contain genetic data that could become part of the DNA during mitosis, potentially leading to alteration in the cell genome.

**Fig. 18:**
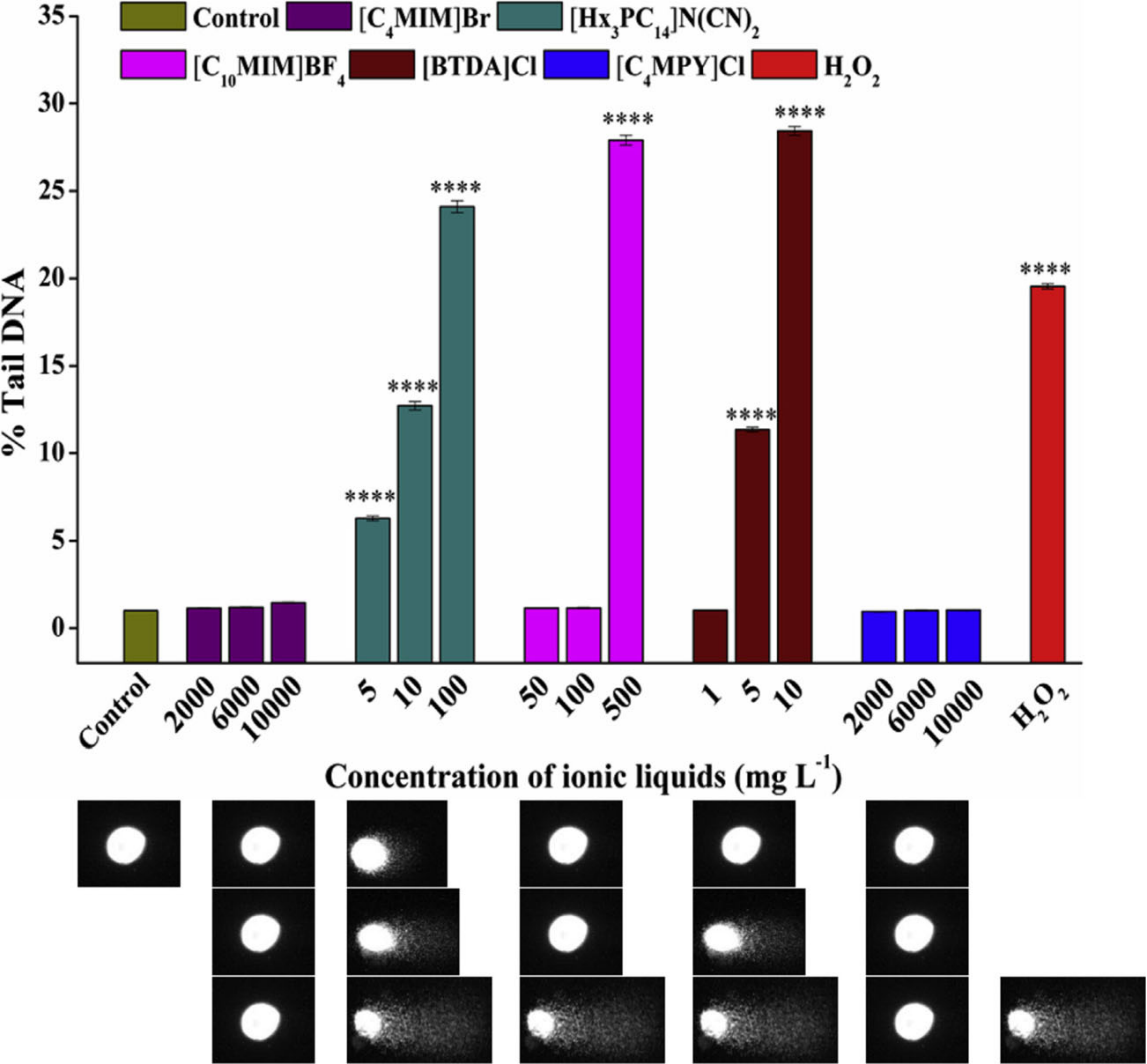
Genotoxicity on MDA-MB-231 cells (expressed in % tail DNA) when treated with different concentration of different ILs compared with control. The representative images of comet formation in the cells are also shown. Figure was taken from Thamke et al. (2019) and reproduced with permission from the publisher

**Fig. 19:**
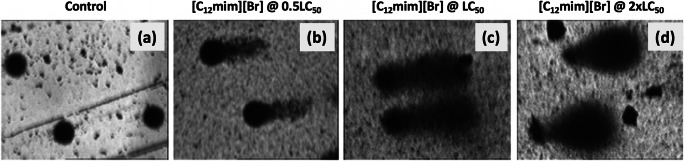
Comet assay for DNA damage. Comets showing tails of different length induced by various concentrations of [C_12_mim][Br] exposure in *U. lactuca*: **a** control; **b** × 0.5 LC_50_; **c** LC_50_; and **d** × 2 LC_50_. LC_50_ stays for lethal concentration, and it is the IL-concentration that causes the death of 50% of the cells. Adapted from Kumar et al. (2011) and reproduced with permission from the publisher

More specifically, DNA fragmentation and damage by ILs have been observed in:

- PC12 cells incubated in [C_8_mim][Br] (Li et al. 2012a) and [C_8_mim][Cl] (Li et al. 2012b).

- A431 cells incubated in [C_17_mim][Cl] (Malhotra et al. 2014).

- MDA-MB-231 cells incubated in [C_10_mim][BF_4_] and [BTDA][Cl] (Thamke et al. 2019), see Fig. 18.
- HepG2 cells incubated in [C_16_mim][Cl] (Wan et al. 2018).

- Human blood lymphocytes cells (Thamke et al. 2017) and in guppy fish *Poecilia reticulata* liver cells (Thamke and Kodam 2016) incubated in [C_10_mim][BF_4_] and [BTDA][Cl].

- CCO cells incubated in [C_4_mim]-based ILs (Radošević et al. 2013).

- DNA damage has also been observed in insect, algae, and plants. For example, DNA fragmentation has been observed in Sf-9 cells following incubation in [C_2_mim][Br] (Wu et al. 2018).

- DNA damage has been observed in marine macroalga *U. lactuca* incubated in [C_12_mim][Br] (Kumar et al. 2011), see Fig. 19.

- DNA damage has been reported for the plant *V. faba* incubated in imidazolium-based ILs (Xu et al. 2020; Liu et al. 2015a; Liu et al. 2016).

- On the good side of DNA-IL interaction, it has been shown how some ammonium-based ILs, which are not toxic against HeLa and K562 cells, can be then used as gene delivery vehicles since they could electrostatically interact with negatively charged DNA or RNA molecules (Bachowska et al. 2012).

### ILs and cell nuclei:

ILs could also find their way to the cell nucleus and either disrupt it or diffuse into it reaching the DNA. The disruption of the nucleus membrane is also a key step in the cell cycle, for instance alteration in its viscoelasticity and integrity could compromise the life of the cell as well as its ability to (properly) replicate.

**Fig. 20:**
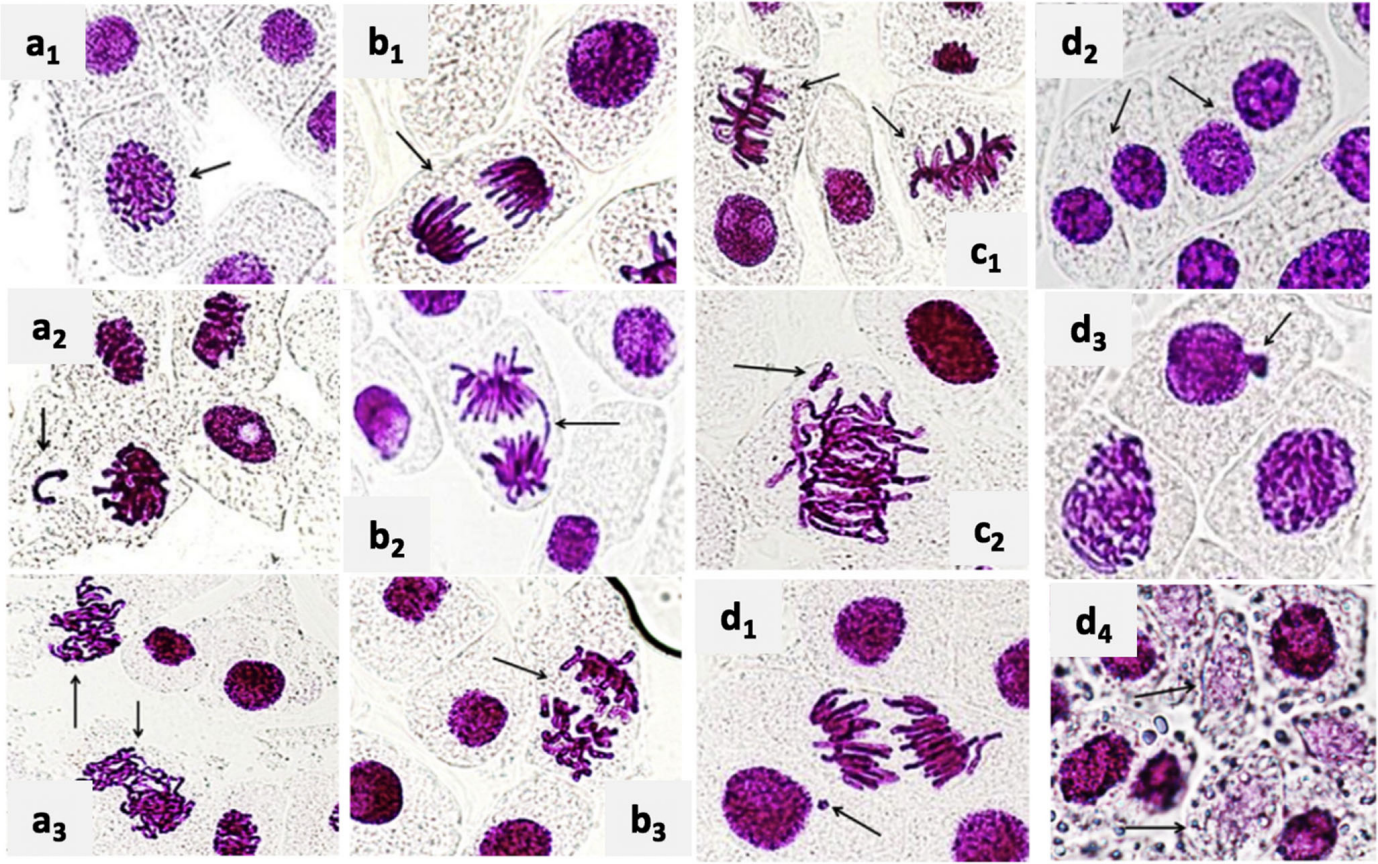
Representative images of different abnormalities observed in *Allium cepa* cells incubated in [C_4_mim][Br], [C_10_mim][BF4], and [BTDA][Cl] ILs. (a_1_) Normal prophase. (a_2_) Prophase with chromosomal loss. (a_3_) Abnormal prophase. (b_1_) Normal anaphase. (b_2_) Anaphase bridge. (b_3_) Abnormal anaphase. (c_1_) Normal metaphase. (c_2_) Metaphase with chromosomal break. (d_1_) Micronuclei formation. (d_2_) Bi-nucleated cells. (d_3_) Nuclear bud. (d_4_) Genetic material loss. Adapted from Thamke et al. (2017) and reproduced with permission from the publisher

Several of these MoAs of ILs have been observed, more specifically:

- Shrinkage of cell nuclei and shape changing to elliptical has been observed in PC12 cells incubated in [C_8_mim][Br] (Li et al. 2012a).

- Diffusion into the cell nuclei followed by DNA damage have been observed in PC12 cells incubated in [C_8_mim][Cl] (Li et al. 2012b).

- Nucleolus-shape alteration and nucleolus-penetration has been observed in CCO cells incubated in [C_n_mim]-based ILs (Radošević et al. 2013).

- Fragmented nuclei and chromatin condensation has been observed in QGY-7701 cells incubated in [C_8_mim][Cl] (Jing et al. 2013).

- Nuclear fragmentation has been observed in NIH/3T3 cells incubated in [C_4_mim][Cl] and [C_4_mim][Gly] (Egorova et al. 2015).

- Breakdown of the nuclear membrane has been observed in Sf-9 cells following incubation in [C_2_mim][Br] (Wu et al. 2018).

- Chromosome breaks, adherence, bridges, multipolar anaphase, C-metaphase, nuclear bud, bi-nucleated cells, polyploidy cells, delayed chromosome, and genetic material loss have been observed in *Allium cepa* cells incubated in [C_4_mim][Br], [C_10_mim][BF_4_], and [BTDA][Cl] (Thamke et al. 2017), see Fig. 20.

### ILs and cell cycle

A consistent set of other studies/findings focuses on the effect of ILs on cell cycle and cell death pathways, determining whether the IL-induced cell death is occurring via apoptosis or necrosis. Obviously, specific MoAs of ILs, as the ones discussed above, are ultimately responsible for cell death. Several flow cytometry experiments have been used for these investigations in which, for example, annexin V assays have been used to discriminate between apoptotic and necrotic cell death, and propidium iodide dye assays have been adopted for cell cycle investigations. Below, a list of several representative studies is reported to give to the reader a glimpse of these investigations as well.

**Fig. 21:**
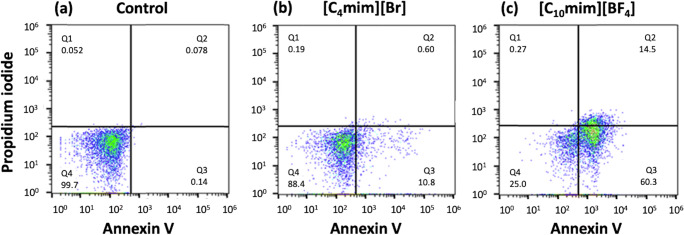
Flow cytometry analysis of apoptosis rates in MDA-MB-231 cells treated with two different imidazolium-based ILs. Adapted from Thamke et al. (2019) and reproduced with permission from the publisher

**Fig. 22:**
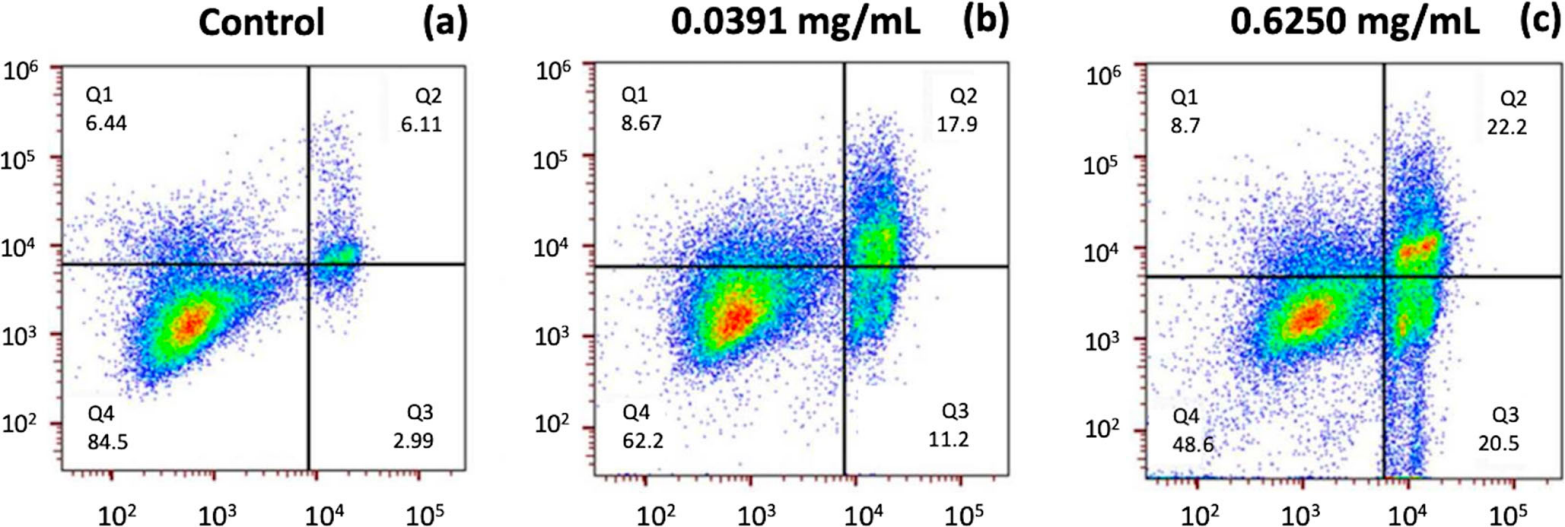
Flow cytometry analysis of apoptosis rates in Sf-9 cells after 24 h of treatment with various concentrations of [C_2_mim][Br]. Adapted from Wu et al. (2018) and reproduced with permission from the publisher.

- Cell cycle arrest at sub-G0 phase has been reported in A431 cells incubated in [C_17_mim][Cl] ILs (Malhotra et al. 2014).

- Reduction of the number of cells in the G1 phase and increment in the S phase has been observed in HEK-293 and rat C6 glioma cells incubated in a piperazinium-based ILs, [C_2_pi][BF_4_] (Yu et al. 2016a).

- Cell death via necrosis has been observed in HaCaT cells after incubation in [BMPY][TFSI] (Hwang et al. 2018).

- Early apoptosis in MDA-MB-231 cells has been observed after incubation in [C_4_mim][Br] and [C_10_mim][BF_4_] (Thamke et al. 2019), see Fig. 21. Both ILs also induce a significant decrease in the G2 and S phases. Interestingly, this study indicates that long alkyl chain ILs affect S and G2 phases at lower concentration as compared with short alkyl chain ILs.

- Apoptosis has been observed in HepG2 cells incubated in [C_8_mim][Br] (Li et al. 2015).
- Increase in the amount of cells at G2/M phase, leading to G2/M phase arrest, have been observed in HepG2 cells incubated in [C_16_mim][Cl] (Wan et al. 2018).

- Apoptosis has been observed in PC12 cells (Li et al. 2012a) and QGY-7701 cells (Jing et al. 2013) incubated in [C_8_mim][Br].

- Apoptosis has been observed in NIH/3T3 cells incubated in [C_4_mim][Cl] and [C_4_mim][Gly] (Egorova et al. 2015).

- Apoptosis has been observed in T47D cells incubated in a series of newly synthesized imidazolium-tethered benzothiazole moieties with fluorinated counter anion ILs (Al-blewi et al. 2019). Interestingly, none of the targeted compound was able to permeate through the blood-brain barrier.

- Early apoptosis in Sf-9 cells has also been observed after incubation in [C_2_mim][Br], together with a significant decrease in G2 and S phases (Wu et al. 2018), see Fig. 22. It has been suggested that early and late apoptosis are the main mechanism at lower and higher IL-concentrations, respectively. At even higher IL-concentrations, on the other hand, no variation in the cell cycle has been observed, suggesting that higher doses of ILs induce necrosis.

### “System biology” of ILs

**Fig. 23:**
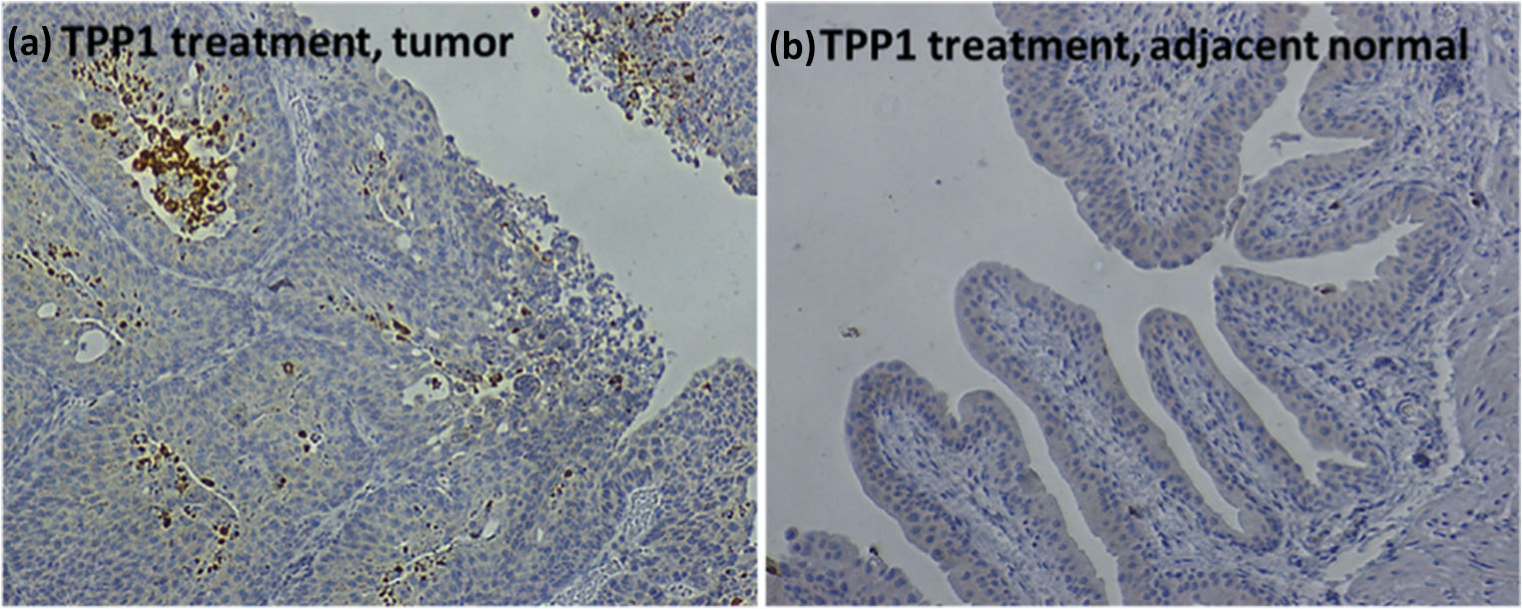
Mouse bladder immunohistology with anti-caspase-3 antibody (dark brown) after treatment with TPP1 on BBN-induced tumors. **a** Area of bladder urothelium with tumor. **b** Sections of tumor-adjacent normal urothelium area clearly showing that the IL selectively kills cancer cells leaving healthy cells unaffected. Adapted from Stromyer et al. (2020) and reproduced with permission from the publisher

**Fig. 24:**
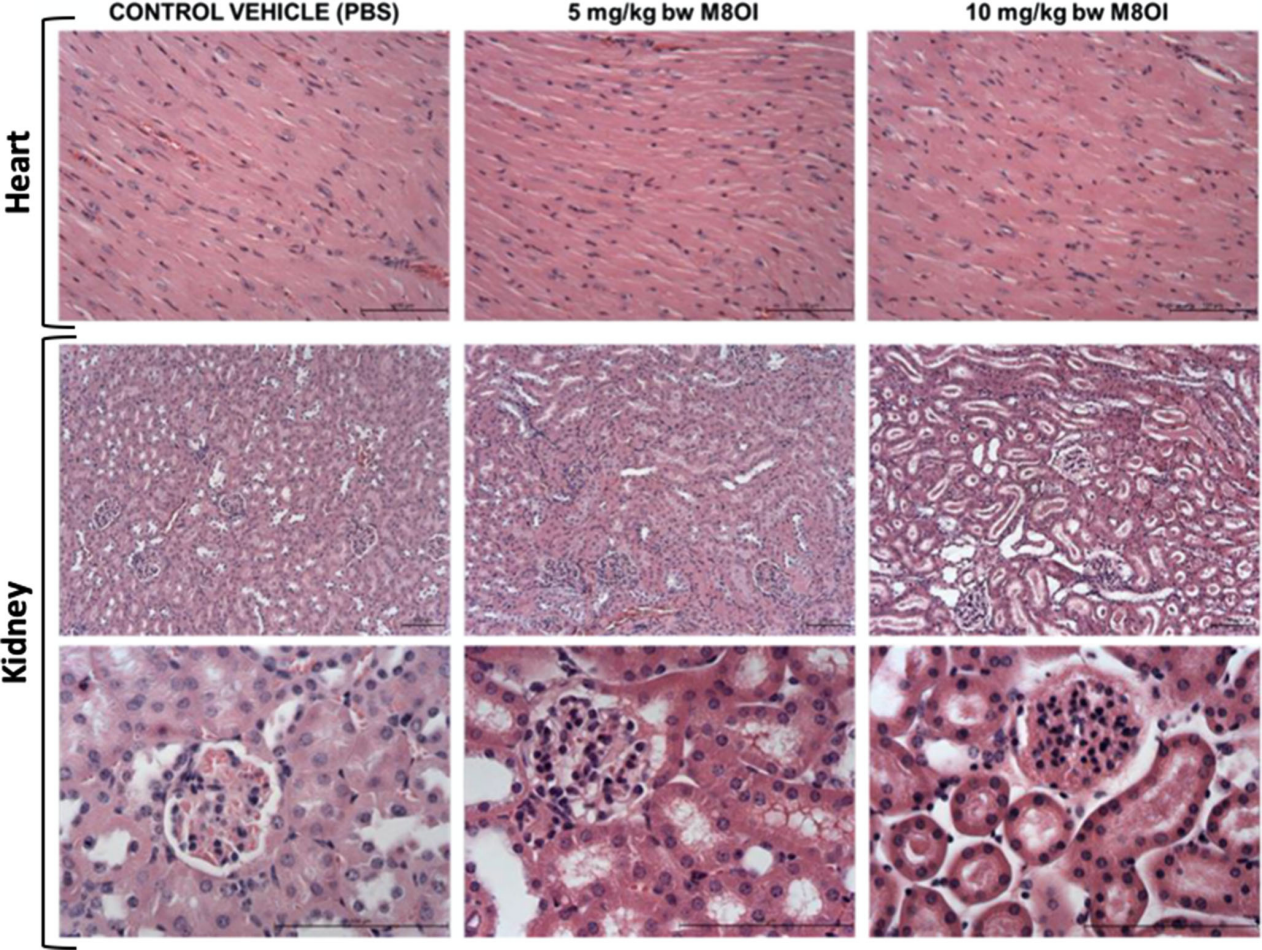
Typical histopathological views of mouse heart (first row) and of mouse kidney (second and third rows), hematoxylin and eosin–stained sections. No changes are visible in the heart tissue, but the IL affect the morphology of the kidney tissue for which morphological changes in both the glomerular and duct regions can be seen. Adapted from Leitch et al. (2020b) and reproduced with permission from the publisher

The MoAs presented up to here are all based on in vitro studies, apart for few cases mainly on plants. The development of actual applications of ILs in nanomedicine, however, requires also the knowledge of how organisms process ILs. ILs can be delivered to organisms in different ways, including oral delivery and injection through the blood stream. ILs can also interact with the skin and get transdermally absorbed as well. At the moment, however, the system biology investigations of ILs are limited, but the few studies reported in the literature somehow confirm that the effect observed in vitro are relevant at in vivo level as well. For example:

- Liver damage, increase in hepatosomatic index, and modified activities of defense enzymes (CAT, GPX, and GST) have been observed in mouse liver exposed to intraperitoneal injection of [C_8_mim][Br] (Yu et al. 2008).
- Marked changes in the gut microbiome have been observed in mice exposed to imidazolium-based ILs via drinking water; however, only mild hepatic and renal effects were observed (Young et al. 2020).

- As a system biology potential application of ILs, it has been shown that in rats, oral delivery of a choline-based IL prevent the absorption of fat molecules through intestinal tissue, potentially providing a feeling of satiety (Nurunnabi et al. 2019). Biochemistry and histology results indicated that the choline-based IL used in the study was tolerated by the rats.

- It has also been shown that a choline-based IL enhances paracellular transport of insulin: a sustained decrease in blood glucose was observed following oral delivery of insulin-IL capsules in rats (Banerjee et al. 2018).

- Exfoliation of the surface layer of the colon, alveolar septa in lung parenchyma, reduced body weight gain, slightly reduced food consumption, slight hematology changes, and statistically significant changes in clinical chemistry parameters (increases in the ALT, SDH, ALP, and GGT activities, and in glucose, blood urea nitrogen, and creatinine concentrations) have been observed in rat exposed to dodecyl-dimethyl-ammonium saccharinate (Jodynis-Liebert et al. 2010).

- Reduction of fetal weight and malformations have been observed in CD-1 mice treated with [C_4_mim][Cl] (Bailey et al. 2008).

- A new imidazolium IL with a triphenylphosphonium substituent (TPP1), applied intravesically to a bladder cancer mouse model induced by N-butyl-N-(4-hydroxybutyl)nitrosamine (BBN), has selectively killed cancer cells leaving healthy cells unaffected as BBN-induced tumors exhibited apoptosis but normal adjacent urothelium did not (Stromyer et al. 2020), see Fig. 23.
- Adult male C57Bl6 mice were acutely exposed to 0–10 mg/kg body weight ionic liquid 1-octyl-3-methylimidazolium (M8OI) via 2 intraperitoneal injections at 0 h and 18 h. After 24 h, dose-dependent degeneration was observed in the kidney as well as mild cholangiopathic changes in the liver; no pathological changes were observed, instead, in the heart and brain (Leitch et al. 2020b), see Fig. 24.

- Hepatopancreas, intestine, and kidney damages have been observed in adult goldfish due to [C_8_mim][Br] exposure, indicating that these are possible target organs (Li et al. 2012c). Subsequent biochemical assays show that [C_8_mim][Br] also induces changes in the activities of the superoxide dismutase, catalase, glutathione peroxidase, malondialdehyde, and glutathione content of hepatopancreas, inducing oxidant stress and lipid peroxidation in hepatopancreas.

- Decrease in antioxidant enzyme activities, DNA damage, production of excess ROS, and increase in malondialdehyde content have been observed in zebrafish liver exposed to [C_10_mim][Br] (Dong et al. 2013).

- Cardiotoxic and hepatotoxic effects induced by ILs have been observed in zebrafish, suggesting potential unanticipated effects on (human) health (Pandey et al. 2017).

- Change in the levels of antioxidant enzymes, glutathione, and malondialdehyde has been observed in earthworm *Eisenia foetida* (*E. foetida*) exposed to [C_8_mim][Br], suggesting cellular lipid peroxidation and formation of ROS in earthworms (Li et al. 2010).

- Inhibition of the activities of Na^+^–K^+^–ATPase, Mg^++^–ATPase, and acetylcholinesterase and variation in cellulase content have been observed in the earthworm *E. foetida* after of exposure to [C_8_mim][Br] (Luo et al. 2009; Luo et al. 2010). The experimental results suggested that the IL could interfere with the nervous function of the earthworms.

- Increase in root membrane permeability and malondialdehyde content and decrease in the antioxidant enzyme activity in roots and leaves have been observed in rice seedlings exposed to [C_8_mim][Cl], which also affect the cellular structures, such as chloroplasts, mitochondria, and rough endoplasmic reticulum (Liu et al. 2013).

- Increase in the activities of antioxidant defense enzymes (superoxide dismutase, catalase, glutathione peroxidase, and glutathione S-transferase) and in levels of the antioxidant glutathione and lipid peroxidation have been observed in *Daphnia magna* exposed to imidazolium-based ILs (Yu et al. 2009).

- Moreover, ILs have been used *in tandem* with drugs in several in vivo studies. For example, it has been shown that an IL-mediated paclitaxel formulation administered intravenously to C57BL/6 mice has a similar antitumor activity and systemic circulation time, slower elimination rate, and significantly smaller hypersensitivity effect compared with cremophor EL-mediated paclitaxel (Chowdhury et al. 2019).

- Topical delivery of siRNA using ILs capable of complexing with siRNA non-covalently and delivering it effectively into skin tested in vivo to SKH-1E hairless mice has significantly suppressed GAPDH expression with no clinical evidence of toxicity (Dharamdasani et al. 2020).

## Final considerations and remarks for the future

**Fig. 25:**
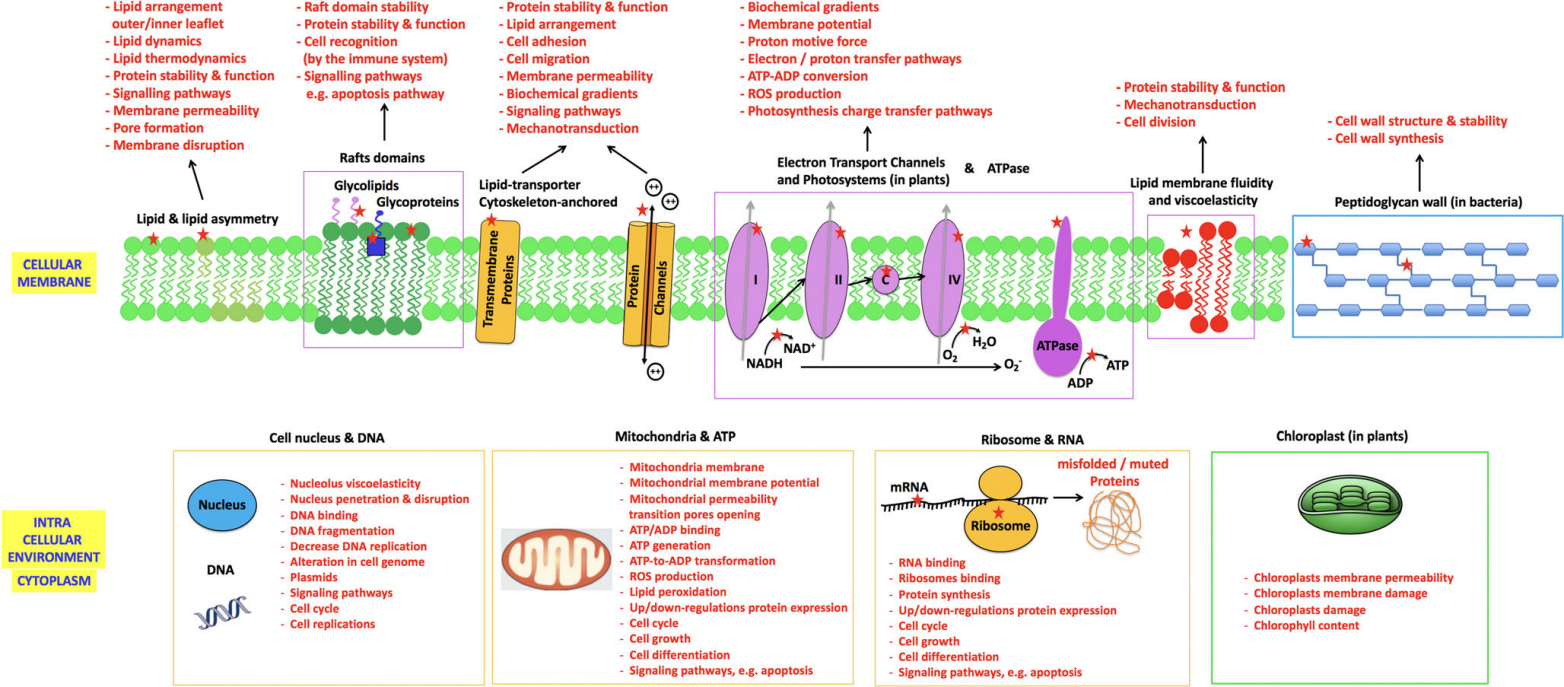
Sketch summarizing the main MoAs of ILs towards living cells presented in this review. The red star stays for ILs

In the above paragraph, the state of the art of the MoAs of ILs towards cells has been presented together with few system biology studies. Overall, it is not always possible to determine whether a measured effect/MoA is directly induced by the ILs or is just a consequence of some other IL-effect. However, the variety of effects is a clear indication of the ability of these organic electrolytes to interact with cells via a variety of different mechanisms, including alteration of lipid distribution and cell membrane viscoelasticity, cell and nuclear membrane damage, mitochondrial permeabilization and dysfunction, generation of reactive oxygen species, chloroplast damage (in plants), alteration of transmembrane and cytoplasmatic protein/enzyme functions, alteration of signaling pathways, and DNA fragmentation (Fig. 25). The experimental and, in few cases, computational methods used in the reported investigations consist of very standard approaches including fluorescence microscopy, flow cytometry, DNA assays, and enzyme activity measurements. Overall, the study of the MoAs of ILs towards cells comprises about 60 original research works, and about twice is the number of works published on the biophysics and chemical-physics of the interaction between ILs and (model) cell membranes. If we compare these numbers with the huge literature on the interaction between antibiotics and drugs with cells, we can certainly conclude that the IL-research effort is still at its infancy. Comparing those numbers is also a meter to highlight the level of details and knowledge in the field of “ILs and cells.” The comparison can finally be useful to guide the IL community to a set of new investigations, as well as stimulate the curiosity of the antibiotic and drug communities to further explore the impact of ILs in their fields. For example, it has been shown that ILs combined with antibiotics and drugs enhance their delivery across skin layers (Moniruzzaman et al. 2010; Zakrewsky et al. 2014; Tanner et al. 2018; Hattori et al. 2019; Tanner et al. 2019; Qi and Mitragotri 2019). Furthermore, even though there are several studies on both cells and model biomembranes on the insertion of ILs, there are no investigations focusing on the effect of pH on IL-insertion, which can offer, however, a pathway to specificity and selectivity of ILs towards cancer cells, as shown for membrane peptides targeting tumors and other acidic tissues (Andreev et al. 2007). Moreover, the membrane disruption mechanism of ILs need to be further investigated to better understand this MoA at microscopic level, as it has been done for antibiotics, e.g., Ma et al. (2019). Finally, we can also mention that effects on cell genomics induced by ILs have never been investigated; genomics studies, however, can be a very important addition to our knowledge of the effect of ILs on cells, and they can also lead to identify new MoAs of ILs, as in the case of antibiotics (Rees et al. 2016). This review would like to promote new and advanced research in the field as well as propose a change of paradigm moving from the “aim to reduce toxicity” to the “aim to use toxicity” as a meter of affinity for applications and start to focus, for example, on identifying how changes in IL physical-chemistry can either neglect or amplify specific MoAs, offering a molecular handle to act on cell biochemistry and mechanobiology via ILs. In this context, it would also be interesting to investigate the effect of sub-toxic doses of ILs (Kumari et al. 2020) and IL-nanodomains on cell behavior rather than just toxicity. In conclusion, the interaction between ILs and cells offers a vast playground for fundamental research which holds the promise of new applications in bio-nanomedicine and bio-nanotechnology. At the core of this new research field, there is a strong and clear connection between physics, chemistry, and biology, which may lead to the identification of (new) bio-chemical-physical MoAs of ILs, e.g., Kumari et al. 2020. New investigations at more detailed level as in the case of the MoAs of antibiotics and drugs will, hopefully, be inspired by this review and will lead to a more solid and stronger effort of our scientific communities in the field of “ILs, biomolecules, and cells.”
